# *Bifidobacterium animalis* subsp. *lactis* BL-16 fermented *Astragali Radix* (W16) promotes the bone growth of juvenile rats via modulation of IGF-1 and gut microbiota

**DOI:** 10.3389/fmicb.2026.1753788

**Published:** 2026-04-14

**Authors:** Ziyi Liu, Ying Cao, Yanan Yang, Jiale Cheng, Jiacheng Zhang, Tianjiao Cong, Qinghang Zhang, Lei Cui, Feng Cai, Yunfeng Duan, Chongming Wu

**Affiliations:** 1 School of Chinese Materia Medica, Tianjin University of Traditional Chinese Medicine, Tianjin, China; 2 PKUMed-Wisbiom Joint Laboratory for Human Microbiome Research, Beijing, China; 3 State Key Laboratory of Chinese Medicine Modernization, Tianjin, China; 4 Tianjin Key Laboratory of Therapeutic Substance of Traditional Chinese Medicine, Tianjin, China

**Keywords:** *Astragali Radix*, *Bifidobacterium animalis* subsp. *lactis* BL-16, bone growth, IGF-1, postbiotic W16

## Abstract

**Introduction:**

Short stature in children causes physical and psychological issues. Human growth hormone (hGH) is clinically recommended but has side effects. Probiotics and functional foods are promising alternatives. *Astragali Radix*, has growth-promoting potential, and fermentation may enhance its bioactivity.

**Methods:**

*Astragali Radix*, was fermented with *Bifidobacterium animalis* subsp. *lactis* BL-16 to prepare postbiotic W16. Juvenile rats were treated with W16, hGH, or unfermented *Astragali Radix*. UPLC-Q-TOF/MS metabolomic profiling, bone growth-related gene (*igf-1*, *trap*) expression, IGF-1 levels, glucose/insulin concentrations, and gut microbiota were analyzed.

**Results:**

W16 enhanced rat body/bone length similarly to hGH, upregulated igf-1/trap, and increased plasma/liver IGF-1—more effectively than unfermented *Astragali Radix*. Unlike hGH, W16 did not induce insulin resistance. While hGH and *Astragali Radix* enriched *Lactobacillus*, W16 uniquely promoted *Acetivibrio cellulolyticus* (beneficial for bone mineral density). Metabolomic profiling revealed pre- and post-fermentation differences in *Astragali Radix*.

**Discussion:**

Postbiotic W16 promotes bone growth via regulating growth-related genes, IGF-1 signaling, and gut microbiota. Safer than hGH (no insulin resistance), W16 is a promising functional food candidate for improving short stature in children.

## 1 Introduction

Childhood linear growth is driven by chondrogenesis at the skeletal growth plate, which is responsible for bone elongation and development (Nilsson and Baron, 2004). Skeletal dysplasia, a condition affecting bone formation and cartilage growth, results in short stature in children (Laugel-Haushalter et al., 2019). Globally, the prevalence of short stature in children is approximately 1.3%, with a relatively higher prevalence in Chinese children, around 3% (Farquharson and Jefferies, 2000). Although short stature does not pose an immediate threat to life, it significantly impacts the quality of life for affected children. Children with short stature are at a higher risk for psychological disorders, such as low self-esteem, academic difficulties, and social immaturity (Kim and Park, 2009). Furthermore, adults who experienced short stature during childhood often report a lower health-related quality of life, even if they are no longer classified as short in height (Jiang et al., 2023). Currently, human growth hormone (hGH) is commonly used in clinic, but it often poses some side effects such as elevation of blood glucose and insulin levels (Labarta et al., 2020). Therefore, discovering more effective and safe methods to deal with short stature should be essential components of children’s health programs.

The gut microbial community is one of several factors that may contribute to optimal growth and bone development in children. Research by Schwarzer et al. (2016) demonstrated that colonization by gut microbiota significantly promoted postnatal growth in germ-free mice and mitigated the adverse effects of chronic undernutrition. In comparison with normal mice, germ-free mice, which lack gut microbiota, exhibited inhibited body growth and reduced longitudinal bone growth rates (Yan et al., 2016), indicating the involvement of gut microbiota in bone growth and development. Additionally, compelling data has proved that supplementation with probiotics, such as *Lactobacillus rhamnosus* JYLR-005, can markedly promote tibial growth, maintain the morphological structure of chondrocytes, improve tibial growth plate development, and balance calcium and phosphorus levels (Liu et al., 2021). Mechanistically, *Lactobacillus* strains, such as *Lactobacillus paracasei* OFS 0291 and *Lactobacillus fermentum* DR9, were found to upregulate the mRNA expression of insulin-like growth factor 1 (IGF-1) and AMP-activated protein kinase (AMPK)-α2 (AMPK-α2), while downregulating the expression of tartrate-resistant acid phosphatase (TRAP), interleukin-6 (IL-6) and IL-1β in aged rats (Hor et al., 2021). A recent study reported that *Bifidobacterium animalis* subsp*. lactis* 11 significantly enhanced the height of children with Prader–Willi syndrome (PWS) (Liu et al., 2021). Hence, probiotics intervention represents a crucial strategy for enhancing bone growth in children.

*Astragali Radix*, the dried root of *Astragalus membranaceus* (Fisch.) Bge.var.mongholicus (Bge.) Hsiao, is known for its tonifying Qi efficacy. It is sweet in taste and slight warm in nature, with a meridian tropism toward lung and spleen (Yang et al., 2024; Su et al., 2024). It contains abundant chemical components, like polysaccharides, saponins and flavonoids (Chu et al., 2021), making it a popular choice in clinical applications to improve various healthy conditions, including short stature. A multicenter randomized controlled trial demonstrated that supplementation with *Astragali Radix* extract mixture HT042 strikingly promoted height growth in children, particularly those with immature skeleton and shorter stature (Huang et al., 2024). Additionally, BHH10, a traditional Chinese medicine formula with *Astragali Radix* as the monarch drug, significantly increased bone mineral density (BMD) and effectively prevented the reduction in trabecular volume, connection density, trabecular number, thickness, and separation in the total femur and femoral neck (Huh et al., 2015).

Probiotic fermentation technology can elevate the content of probiotic-related enzymes and metabolites, potentially converting traditional Chinese medicine (TCM) components into active functional ingredients. For example, fermenting *Scutellaria baicalensis* with *Lactobacillus brevis* RO1 notably promotes the bioconversion of baicalin and wogonoside to baicalein and wogonin, respectively (Xu and Ji, 2013). Similarly, *Rhizoma Atractylodis Macrocephalae* fermented with *Lactobacillus plantarum* can regulate the gut microbiota and gut permeability, offering greater anti-obesity effects than its unfermented counterpart (Wang et al., 2015). Fermented red ginseng extract has also shown increased effectiveness in inducing apoptotic cell death and preventing cancer stem cell differentiation compared to unfermented red ginseng (Oh et al., 2015). Furthermore, *Astragali Radix* polysaccharides fermented with *Lactobacillus acidophilus* can serve as a functional food due to its better effect on promoting calcium absorption and alleviating osteoporosis (Zhou et al., 2023). Although these progresses have yielded, the effect of fermented *Astragali Radix* on bone development using another probiotic, *Bifidobacterium* strain, remains unexplored.

As such, we unpacked this study to assess the effects of *Bifidobacterium animalis* subsp. *lactis* BL-16 fermented *Astragali Radix* on bone growth and development, comparing it to the effects of unfermented *Astragali Radix*. Our research aims to advance the application of probiotic fermentation technology in traditional Chinese medicine (TCM) and provide insights into developing interventions to promote bone growth in children with short stature.

## 2 Materials and methods

### 2.1 Materials

The breast milk-derived strain BL-16 was identified as *Bifidobacterium animalis* subsp. *lactis* through 16S rDNA sequencing, and has been deposited in China General Microbiological Culture Collection Center (CGMCC) under accession number CGMCC No. 32050. Whole-genome sequencing and functional prediction analysis revealed a high similarity between BL-16 and BL-11, a strain that has undergone clinical trials for promoting children’s growth and development. However, compared to BL-11, BL-16 requires a shorter fermentation time and achieves a higher fermentation density when used for fermentation. Therefore, *Bifidobacterium animalis* subsp. *lactis* BL-16 was selected as the strain for fermentation in this study.

Fermentation substrate (FS) used in this study was a mixture (1:1) of a basal medium and *Astragali Radix* extract. The basal medium includes peptone, yeast extract, glucose (C_6_H_12_O_6_·H_2_O), magnesium sulfate (MgSO_4_·7H_2_O), sodium acetate (CH_3_COONa·3H_2_O), ammonium citrate [(NH_4_)_2_HC_6_H_5_O_7_], and dipotassium phosphate (K_2_HPO_4_·3H_2_O).

### 2.2 Preparation of *Astragali Radix* extract and BL-16 postbiotics (W16)

The *Astragali Radix* extract was prepared by weighing an appropriate amount of *Astragali Radix* powder (Tong Ren Tang Co., Ltd., Beijing, China). Add 10 volumes of distilled water and extract for 1 h, then filter. The filter residue is extracted again with 10 volumes of distilled water for 45 min. The filtrate is then concentrated to obtain the *Astragali Radix* extract (1 g/mL), dilute with MRS medium (Peptone 10.0 g/L, beef extract 8.0 g/L, yeast extract 4.0 g/L, glucose 20.0 g/L, dipotassium hydrogen phosphate 2.0 g/L, diammonium hydrogen citrate 2.0 g/L, sodium acetate 5.0 g/L, magnesium sulfate 0.2 g/L, manganese sulfate 0.04 g/L, Tween 80 1.0 g/L, and cysteine 0.5 g/L) to 1 g/mL and set aside.

The BL-16 strain was revived and passaged twice for activation. A 10% inoculum was then introduced into the fermentation substrate (FS) and incubated anaerobically at 37 °C for 36 h. After fermentation, the culture was pasteurized to produce BL-16 postbiotics (WISYNBIO-16, abbreviated as W16). After fermentation, the culture was centrifuged at 4,000 r/min for 20 min, and the supernatant was collected as the test sample and stored at 4 °C for subsequent experiments.

### 2.3 Animal experiment

All experimental procedures strictly adhered to the guidelines for the care and use of laboratory animals established by the National Institutes of Health (NIH). The animal experiment was ethically approved by Tianjin University of Traditional Chinese medicine (No. TCM-LAEC2023176). All methods and procedures adhered to established animal welfare guidelines.

Thirty-two three-weeks old male SD rats were purchased from Sibeifu Beijing Biotechnology Co. Ltd. (Beijing, China). The animals were raised in a specific pathogen free (SPF) facility with 12/12-h light cycle, room temperature 22 ± 1 °C, humidity 60%, and free access to food and water. After seven-day adaptive feeding, they were randomly divided into four groups with eight rats in each group: normal control group (NC, orally administrated with saline), human growth hormone group (hGH, daily subcutaneous injections of hGH, 1 IU/kg), fermentation substrate group (FS, orally administrated with fermentation substrate of *Astragali Radix*, 500 mg/kg), BL-16 postbiotics group (W16, orally administrated with W16, 500 mg/kg). During experiment, the body weight of each rat was recorded. After 14 days treatment, the rats were anesthetized and their blood samples were collected by removing eyeballs. The serum samples were obtained by centrifugation for 10 min at 5,000 rpm at 4 °C. The brains were isolated after transcardial perfusion with saline. Simultaneously, the cecal tissue, tibia and sections 3, 4, and 5 of the spine were collected and stored at −80 °C.

### 2.4 Measurement of body length

The body length of each rat was measured using ruler (cm) on the 1st, 6th, and 11th and 15th days of the experiment. The body length is defined as the distance from the tip of the nose to the anus of a rat. The Lee’s index was calculated by the formula Lee’s index = (weight × 1,000)^(1/3)^/body length (cm).

### 2.5 Measurement of bone length

The extra tissue was removed using forceps and a scalpel blade. Then the intact left and right tibia retaining the upper and lower growth plates was obtained. The length of left and right tibia was measured using ruler (cm).

### 2.6 Micro CT imaging analysis of bone

Micro-CT imaging was performed on the right tibia and fifth lumbar vertebra collected from rats. The 3D reconstruction software was performed using the EVS Beam software. Scanning parameters were as follows: Voltage: 40 KVp, current: 113 μA, Fov: 72 mm. Bone mineral density (BMD) was analyzed using Analyze 12.0 software.

### 2.7 Neurotransmitter analysis of brain tissue

The levels of 12 neurotransmitters, including dopamine, 3-Methoxytyramine, tryptamine, Metanephrine, Histamine, (−)-Norepinephrine, γ-Aminobutyric acid, Tyramine, Serotonin, Agamatine, (±)-Epinephrine and (±)-Octopamine were measured using an UHPLC-O-Exactive-Orbitrap triple quadrupole mass spectrometer (Thermo Fisher Scientific, United States) equipped with a Waters ACQUITY UPLC BEH C8 liquid chromatography column BEH C8 (2.1 × 100 mm, 1.7 μm). The analytic conditions are as follows. The mobile phases A is 0.004% formic acid and 5 mM ammonium bicarbonate and B is 0.16% formic acid and 2 mM ammonium formate. The gradient condition is 0–2 min 7% B, 2–5 min 22% B, 5–8.5 min 30% B, 8.5–8.6 min 45% B, 8.6–12.1 min 95% B, 12.1–15 min 7% B. The flow rate is 0.5 mL/min. The column temperature was 50 °C and injection volume was 1 μL. The MS parameters were as follows: capillary voltage was set at 4,000 V. Source temperature was maintained at 130 °C, while the desolvation temperature was set at 300 °C. N2 was used as the desolvation gas (flow rate of 10 L/min), and Ar was used as the collision gas (flow rate of 0.15 mL/min).

### 2.8 Blood glucose measurement

Blood from 12 h-fasted rat was used to measure fasting blood glucose levels using a commercial kit (Jiancheng, Nanjing, Jiangsu, China) according to manufacturer’s instructions.

### 2.9 ELISA assays

Serum IGF-1, insulin and liver IGF-1 levels were using IGF-1 ELISA Kit and INS ELISA Kit (Jiangsu Meimian Industrial Co., Ltd) following the manufacturer’s instructions.

### 2.10 RNA extraction and real-time quantitative reverse transcription PCR (RT-qPCR)

The total RNA of bone and liver was extracted using the TRIzol Reagent (Thermo Fisher Scientific, 15596026). The relative expression of *igf-1*, *alp* and *trap* was quantified by RT-qPCR assay using specific sense and antisense PCR primers. The primers were as follows: *igf-1*: Forward 5′-GCTTGCTCAC CTTTACCAGC-3′; Reverse 5′-AAGTG TACTTCCTTCTGAGTCT-3′. *alp*: Forward 5′-GACGGTGAACGGGAGAAC-3′; Reverse 5′-GACGGTGAACGGGAGAAC-3′. *trap*: Forward 5′-CGCCAGAACCGTGCAGA-3′; Reverse 5′-TCAGGCTGCTGGCTGAC-3′. β-actin: Forward 5′-CCTGTACGCCAACACAGTGC-3′; Reverse 5′-ATACTCCTGCTTGCTGATCC-3′.

### 2.11 Full length 16S rRNA gene sequencing

Fecal samples were immediately frozen using liquid nitrogen and subsequently stored at −80 °C. Total DNA was extracted using a QIAamp DNA Stool Mini Kit (Qiagen, Valencia, CA, United States) following the method as previous reported (Qiao et al., 2021). The V1-V9 region of 16S rRNA gene was amplified using specific primer with the barcode and PCR reactions were performed with TransStart® FastPfu DNA Polymerase (TransGenBiotech) (Yang Y. et al., 2023). The PCR products were then pooled at equidensity ratios and purified using the QIAquick@GelExtraction Kit (QIAGEN). Following this, the sequencing library was constructed using SMRTbellTM Template Prep Kit (PacBio), in adherence to the manufacturer’s protocol. To evaluate the library’s quality, we utilized the Qubit@ 2.0 Fluorometer (Thermo Scientific) and the FEMTO Pulse system. Sequencing was carried out on the PacBio Sequel platform.

### 2.12 Bioinformatic analysis

Bioinformatic analysis was performed as previously reported (Yang et al., 2022). Briefly, raw sequences were processed using the PacBio SMRT portal for initial analysis, setting a threshold to classify a CCS as noise if it fell below 90% accuracy. Subsequently, amplicons were trimmed on the PacBio platform to discard sequences outside the expected amplicon size range (minLength 1,340 bp, maxLength 1,640 bp). To detect chimeric sequences, the reads were cross-referenced with the UCHIME algorithm^1^ (Edgar et al., 2011). Sequence analysis was performed using the Uparse software (Uparse v7.0.1001, http://drive5.com/uparse/) (Edgar, 2013). Sequences with a similarity of 97% or greater were clustered into the same operational taxonomic units (OTUs). Taxonomic classification was then assigned based on the Mothur algorithm, referencing the SSUrRNA Database of Silva Database (Version 138.1)^2^ (Wang et al., 2007). Measures of both alpha and beta diversity were computed using QIIME software (Version 1.9.1) and visualized with R software (Version 4.2.3). Species showing significant differences were identified based on a *p*-value <0.05 and Fold Change >2.

### 2.13 UPLC-Q-TOF/MS analysis

The analysis was performed using a gradient elution program. Mobile phase A was ultrapure water containing 0.5% formic acid, and mobile phase B was acetonitrile. The gradient program was as follows: 0.0–5.0 min, 5% B; 5.0–13.0 min, 5–35% B; 13.0–17.0 min, 35–45% B; 17.0–26.0 min, 45–85% B; 26.0–27.0 min, 85–100% B; 27.0–28.0 min, 100% B; 28.0–28.5 min, 100–5% B; and 28.5–30.0 min, 5% B for column re-equilibration. The injection volume was 4 μL, the flow rate was maintained at 0.4 mL/min, and the column temperature was set at 35 °C.

Data were acquired in both positive and negative ion modes using an electrospray ionization (ESI) source. The ion source temperature was 600 °C. The nebulizer gas (GS1), auxiliary gas (GS2), and curtain gas pressures were set at 60, 60, and 35 psi, respectively. For full-scan MS^1^ analysis, the mass range was *m*/*z* 50–1,200 with an accumulation time of 0.1 s; the declustering potential (DP) was ±80 V, and the collision energy (CE) was ±10 eV. For MS^2^ acquisition in information-dependent acquisition (IDA) mode, the mass range was *m*/*z* 25–1,000 with an accumulation time of 0.035 s; the DP was ±60 V, and the CE was set to ±40 eV.

### 2.14 Statistical analysis

Analysis of the data was conducted using GraphPad Prism (Version 8.3.0). Independent Student’s t test was used to compare differences between two groups and *p*-value less than 0.05 was considered statistically significant.

## 3 Results

### 3.1 Fermented *Astragali Radix* promotes bone growth

**Figure 1:**
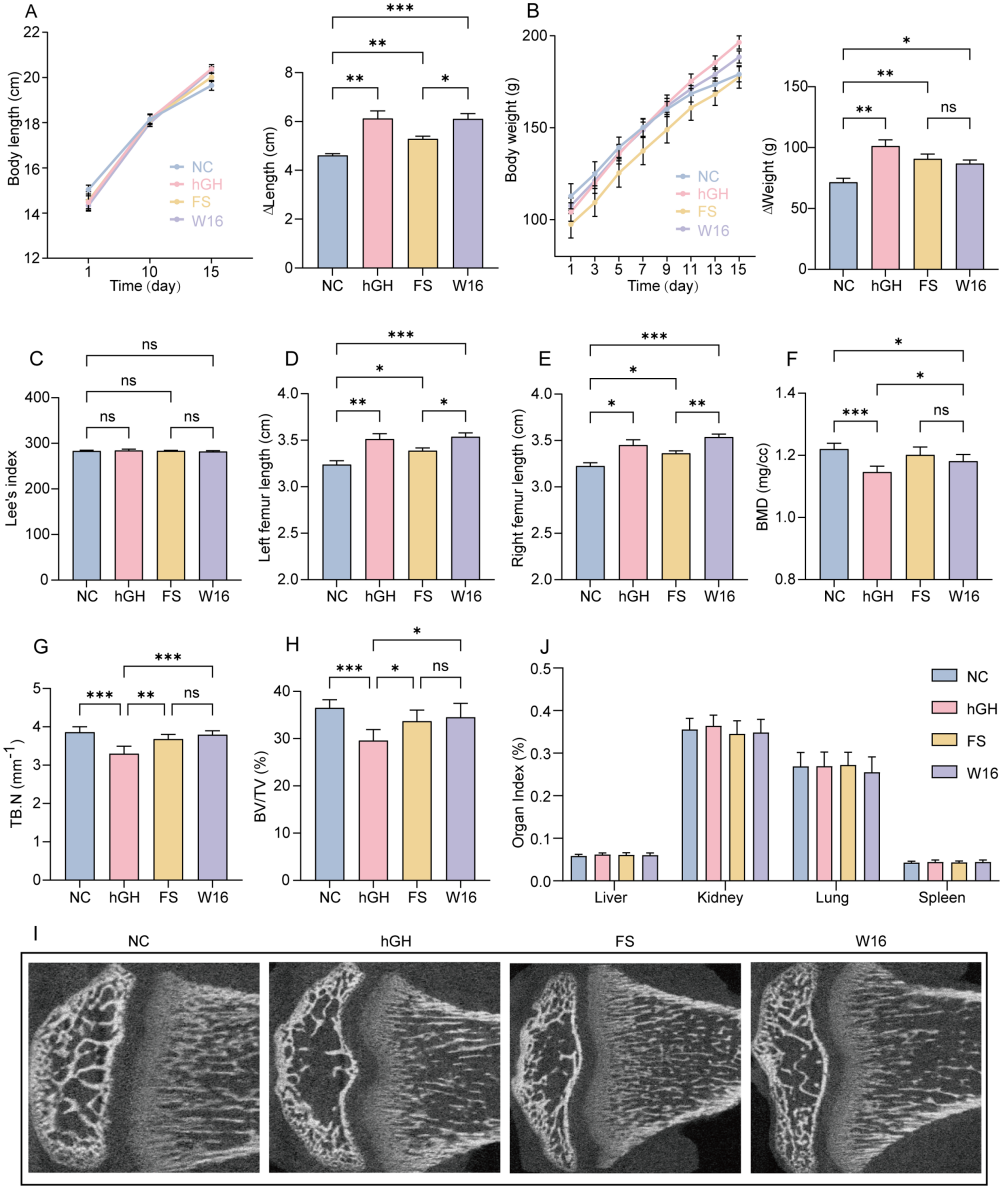
Fermented *Astragali Radix* promotes bone growth. **(A)** Line graph of the body length (cm) and variations of body length (cm). **(B)** Line graph of the body weight (g) and variations of body weight (g). **(C)** Lee’s index of four groups. **(D,E)** Length of left femur (cm) and length of right femur (cm). **(F–I)** Bone mineral density (mg/cc), trabecular bone number (mm^−1^), bone volume/total volume (mm^−1^), and computed tomography images of the tibia. **(J)** Organ indices of the liver, kidney, lung, and spleen of each rat among four groups. ^*^p < 0.05, ^**^p < 0.01, and ^***^p < 0.001.

To explore the function of fermented *Astragali Radix* in skeletal development and growth, we monitored the body length and body weight of each rat throughout the experiment, along with measured the femur length and bone mineral density at the end of experiment. Expectedly, human growth hormone (hGH) obviously promoted the increase of body length in rats after 2 weeks injection. Compared with NC group, unfermented *Astragali Radix* also promoted an increase in body length. Stunningly, following *Bifidobacterium animalis* subsp. *lactis* BL-16 fermentation, *Astragali Radix* exhibited an enhanced effect on body length, similar to that of hGH (Figure 1A). Body weight, another growth indicator, was notably increased by hGH, unfermented *Astragali Radix* and fermented *Astragali Radix*, although the groups treated with *Astragali Radix* showed slightly lower increases than the hGH group (Figure 1B). Lee’s index, used to evaluate obesity in adult rats, showed no significant differences between the four groups (Figure 1C). Both the left and right femur lengths were significantly increased in hGH-treated rats. While unfermented *Astragali Radix* also had a positive effect, fermented *Astragali Radix* demonstrated even greater advantages, with results close to those of hGH (Figures 1D,E). Bone mineral density (BMD), a measure of bone mineralization, was significantly reduced by hGH. Although fermented *Astragali Radix* also caused a decrease, the reduction was notably smaller than that observed with hGH. Similarly, hGH exhibited effects on trabecular bone number (TB.N) and bone volume fraction (BV/TV), while the FS and W16 groups showed no significant changes (Figures 1F–I). These findings indicate that BL-16 fermented *Astragali Radix* effectively promotes bone growth and development. Meanwhile, we weighed the liver, kidney, spleen, and lung tissues of rats in each group and calculated the corresponding organ indices. The results showed no significant differences among the groups, indicating that neither hGH, unfermented *Astragali Radix*, nor BL-16 fermented *Astragali Radix* had adverse effects on the major organs (Figure 1J).

### 3.2 Fermented *Astragali Radix* upregulates bone growth gene expression

**Figure 2:**
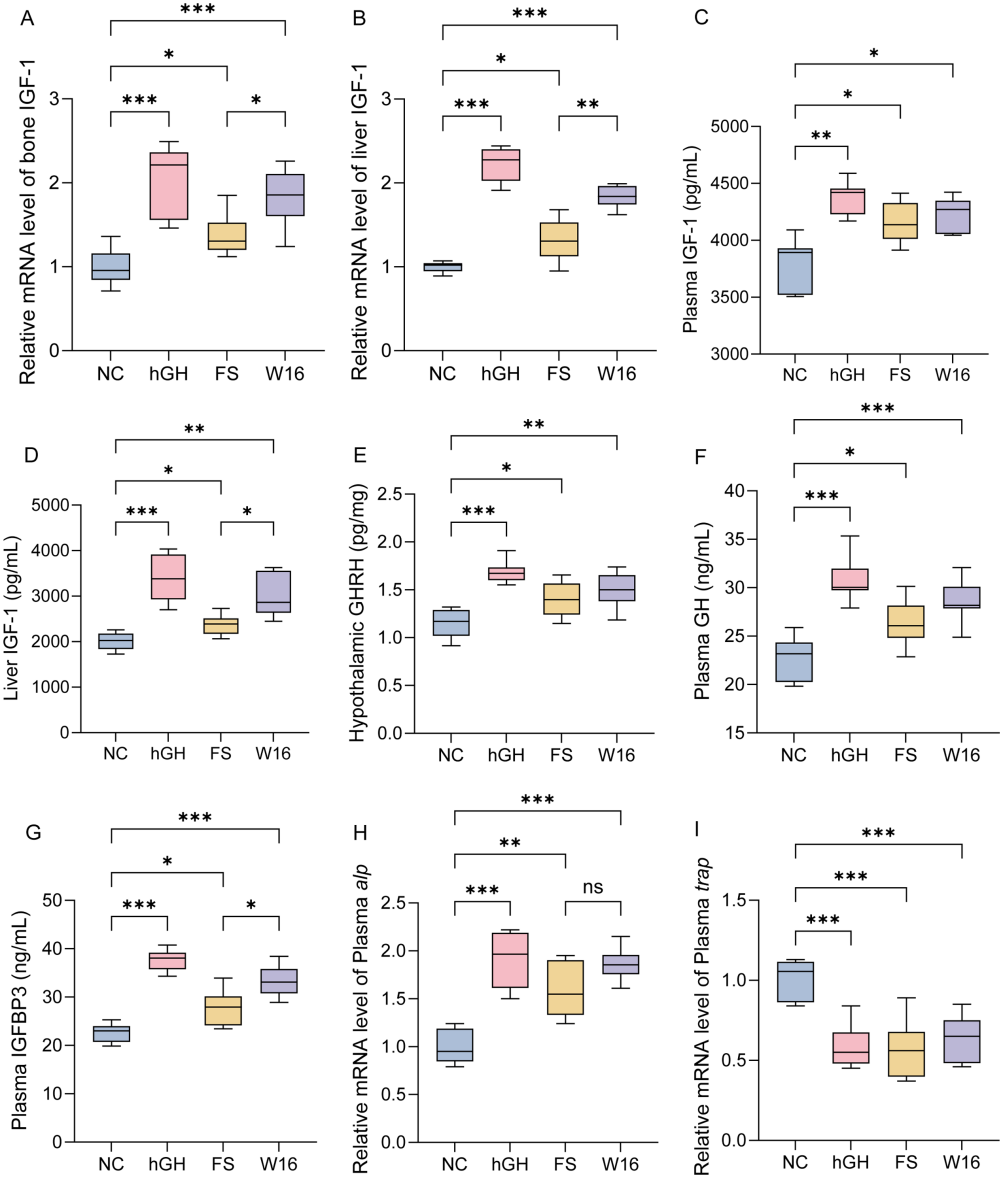
Fermented *Astragali Radix* upregulates bone growth gene expression. **(A)** Relative mRNA level of bone *igf-1*. **(B)** Relative mRNA level of liver *igf-1*. **(C)** Plasma level of IGF-1 (pg/mL). **(D)** Liver level of IGF-1 (pg/mL). **(E)** Relative mRNA level of plasma *alp*. **(F)** Relative mRNA level of plasma *trap*. ^*^p < 0.05, ^**^p < 0.01, and ^***^p < 0.001.

The GH/IGF-1 axis plays a crucial role in bone formation and growth performance. Therefore, we investigated whether fermented Astragalus could regulate the GH/IGF-1 axis. Notably, hGH, unfermented *Astragali Radix* and fermented *Astragali Radix* all upregulated igf-1 gene expression in bone and liver tissues. The effect was ranked from highest to lowest as follows: hGH, fermented *Astragali Radix*, and unfermented *Astragali Radix* (Figures 2A,B). Correspondingly, the plasma and liver levels of IGF-1 also significantly increased following treatment with hGH, fermented *Astragali Radix*, and unfermented *Astragali Radix*, with the BL-16 group showing an enhancement close to that of the hGH group (Figures 2C,D). Subsequently, based on the GH/IGF-1 axis, we measured serum levels of GH and IGFBP-3, as well as hypothalamic GHRH levels. We found that both fermented and unfermented Astragalus could elevate these levels, but the effect of fermented Astragalus was closer to that of hGH (Figures 2E–G). As an early marker of osteogenic differentiation, *alp* gene expression was dramatically enhanced by hGH, unfermented *Astragali Radix* and fermented *Astragali Radix* compared to control rats. The promoting effect of fermented *Astragali Radix* was similar to that of hGH (Figure 2H). Trap, a lytic enzyme responsible for bone resorption and a functional marker of osteoclast cells, showed significantly decreased mRNA levels in bone following treatment with hGH, unfermented *Astragali Radix*, and fermented *Astragali Radix* (Figure 2I). These results indicate that fermented *Astragali Radix* obviously promotes the gene expression related to bone development, thereby enhancing growth in rats.

### 3.3 Fermented *Astragali Radix* alleviates insulin resistance in juvenile rats

**Figure 3:**
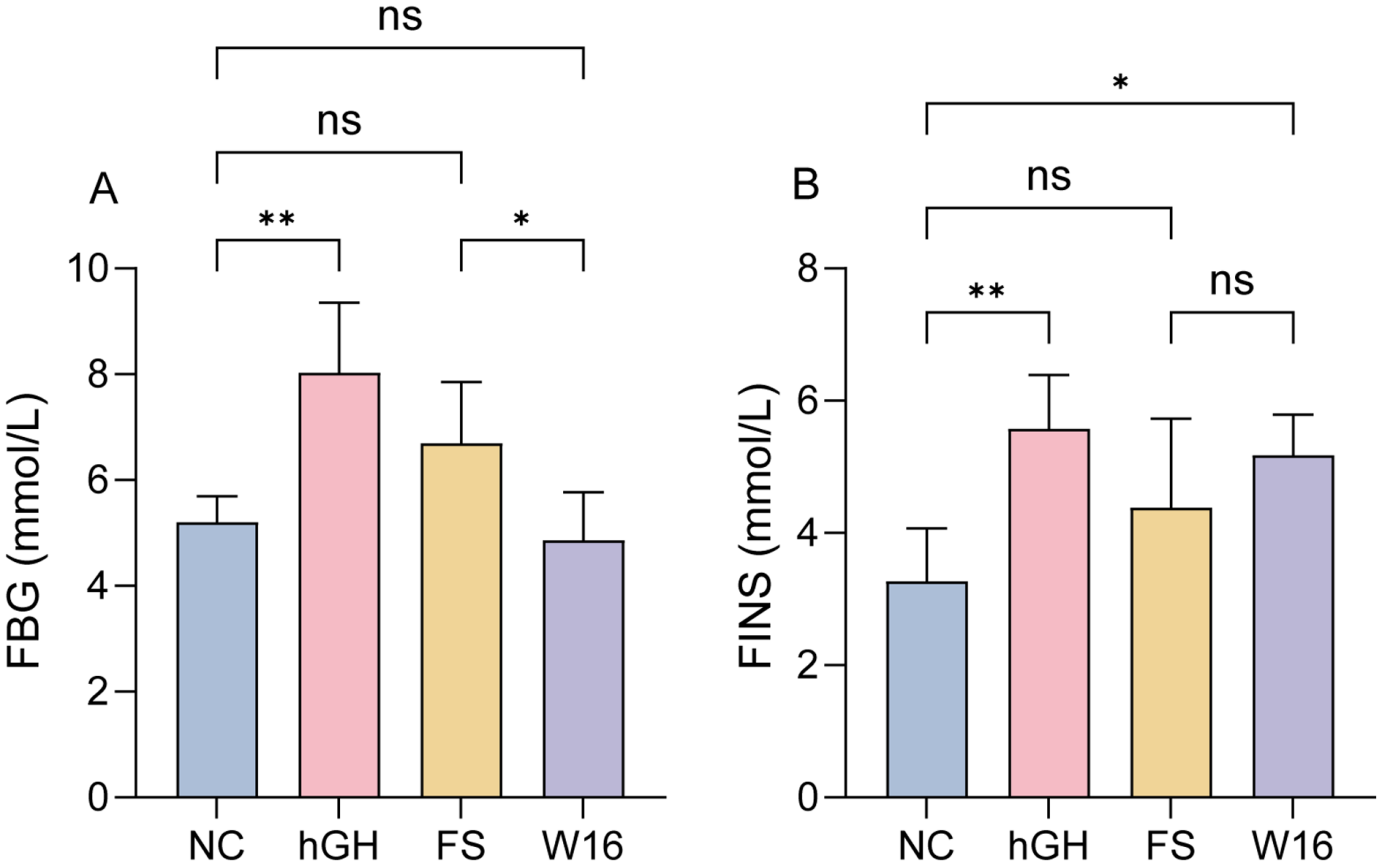
Fermented *Astragali Radix* alleviates insulin resistance of juvenile rats. **(A)** The level of fasting blood glucose (mmol/L). **(B)** The level of serum insulin (mU/L). ^*^p < 0.05 and ^**^p < 0.01.

It is commonly acknowledged that growth hormone can decrease glucose uptake by peripheral tissues and reduce cellular sensitivity to insulin, leading to insulin resistance and elevated blood glucose levels. Consistent with this, hGH notably elevated fasting blood glucose and serum insulin levels compared to the NC group (Figures 3A,B). In contrast, neither unfermented *Astragali Radix* nor fermented *Astragali Radix* affected fasting blood glucose levels. Although fermented *Astragali Radix* did elevate serum insulin, this effect was less pronounced than that of hGH (Figures 3A,B), suggesting that while fermented *Astragali Radix* promotes bone growth, it does not induce the insulin resistance side effectt associated with hGH.

### 3.4 Fermented *Astragali Radix* alters the neurotransmitter levels of juvenile rats

**Figure 4:**
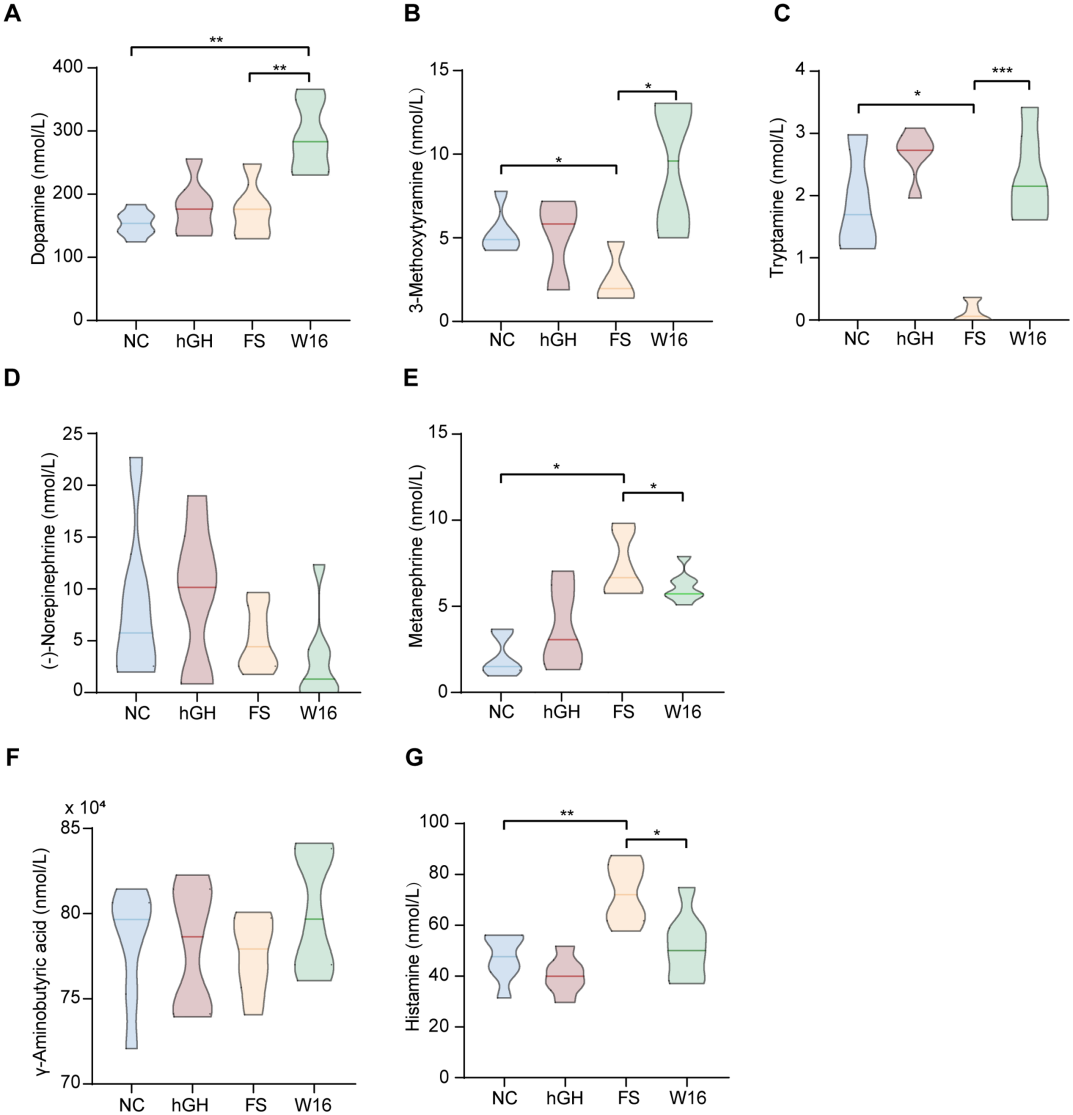
Fermented *Astragali Radix* alters the neurotransmitter levels of juvenile rats. The level of **(A)** dopamine, **(B)** 3-methoxytyramine, **(C)** tryptamine, **(D)** (−)-norepinephrine, **(E)** metanephrine, **(F)** γ-aminobutyric acid, **(G)** histamine in hippocampus tissue. ^*^p < 0.05, ^**^p < 0.01, and ^***^p < 0.001.

Co-secretion of hGH with neurotransmitters suggests that neurotransmitters can affect the secretion of growth hormone. We next detected the levels of 12 neurotransmitters across four groups. Only 7 out of 12 neurotransmitters assayed were detectable. Overall, hGH exerted no effect on these seven neurotransmitters. Notably, dopamine levels were significantly elevated by fermented *Astragali Radix* (Figure 4A). Additionally, 3-Methoxytyramine and tryptamine levels were notably reduced by unfermented *Astragali Radix*, but fermented *Astragali Radix* administration strikingly reversed these changes (Figures 4B,C). Conversely, Metanephrine and Histamine were significantly increased by unfermented *Astragali Radix*, but fermented *Astragali Radix* markedly decreased their levels (Figures 4E,G). Levels of (−)-Norepinephrine and γ-Aminobutyric acid did not differ significantly among the four groups (Figures 4D,F).

### 3.5 Fermented *Astragali Radix* alters the gut microbiota of juvenile rats

**Figure 5:**
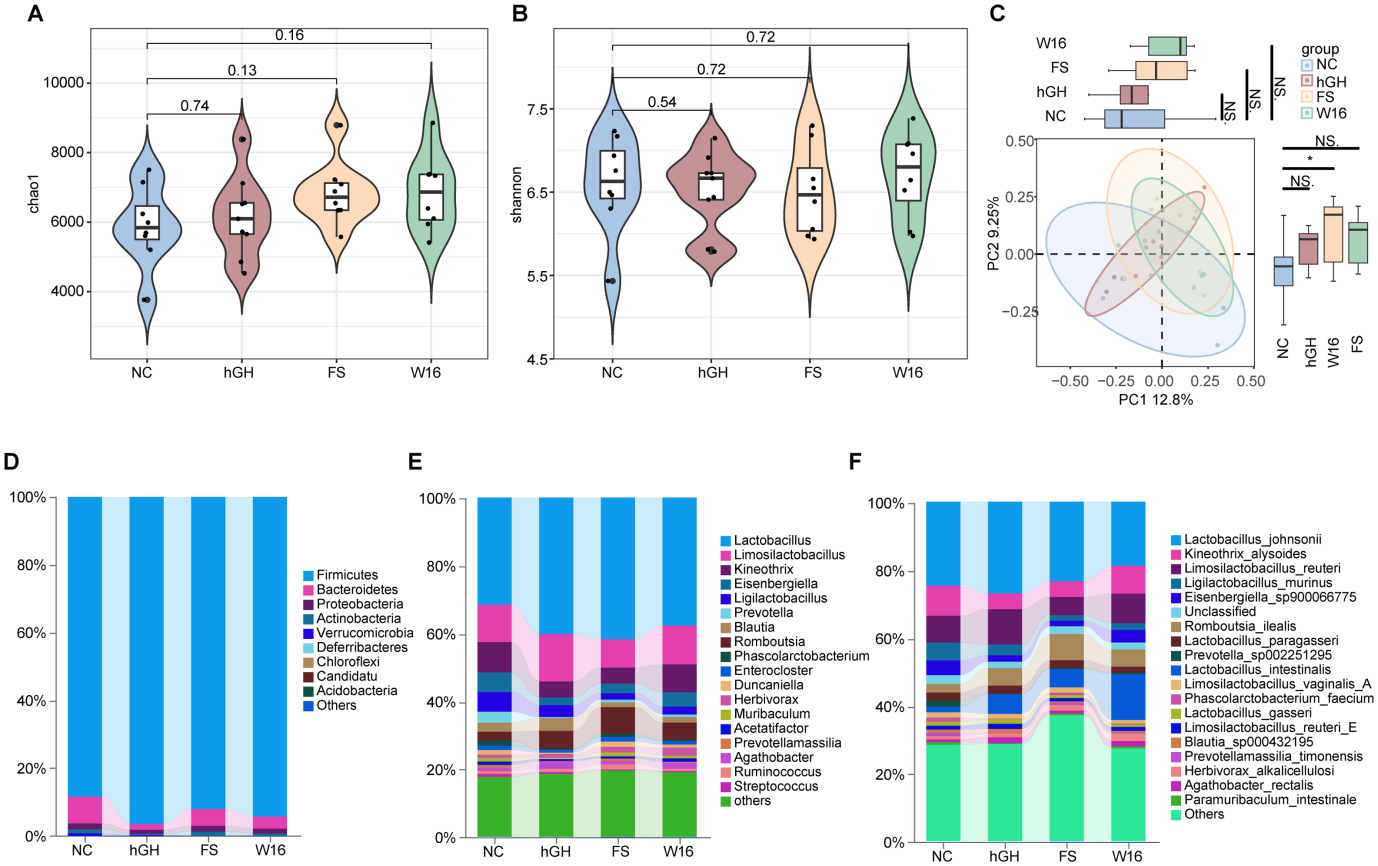
The diversity and composition of gut microbiota in juvenile rats. The alpha-diversity of gut microbiota was assessed by **(A)** Chao 1 and **(B)** Shannon index. **(C)** Principal coordinates analysis (PCoA) of microbiome beta diversity. Taxonomic distributions of bacteria at the **(D)** phylum, **(E)** genus, and **(F)** species level among four groups.

The existence of gut-bone axis indicates that the gut microbiome is a crucial regulatory factor affecting bone homeostasis. Therefore, we compared the alterations in gut microbiota composition of juvenile rats following different treatment. Alpha diversity, indicated by Chao1 and Shannon indices, did not significantly change among the four groups (Figures 5A,B). Principal coordinate analysis (PCoA) showed that the microbial structure was not significantly different among NC, hGH and Am groups. Only the second principal component (PC2) varied significantly between the NC and BL-16 groups (Figure 5C). We then compared the taxonomic profile of the gut microbiota at the phylum, genus and species level, respectively. The five most phyla were Firmicutes, Bacteroidetes, Proteobacteria, Actinobacteria and Verrucomicrobia. Firmicutes increased, while Bacteroidetes and Actinobacteria decreased post hGH treatment. These alterations were obviously reversed by both unfermented and fermented *Astragali Radix* (Figure 5D). At the genus level, compared with NC group, *Lactobacillus* increased with hGH and unfermented *Astragali Radix*, but was further elevated by fermented *Astragali Radix*. *Eisenbergiella* showed the opposite trend, decreasing in the hGH and Am groups, but increasing in the BL-16group. *Romboutsia* increased in all three treatment groups compared to NC group. *Ligilactobacillus* and *Blautia* decreased after unfermented and fermented *Astragali Radix* treatment compared to the NC and hGH groups (Figure 5E). Comparative analysis highlighted that hGH, unfermented and fermented *Astragali Radix* treatments amplified species like *Romboutsia ilealis* and *Lactobacillus intestinalis*, whereas suppressing the growth of *Ligilacttobacillus murinus* and *Prevotella sp002251295* (Figure 5F).

**Figure 6:**
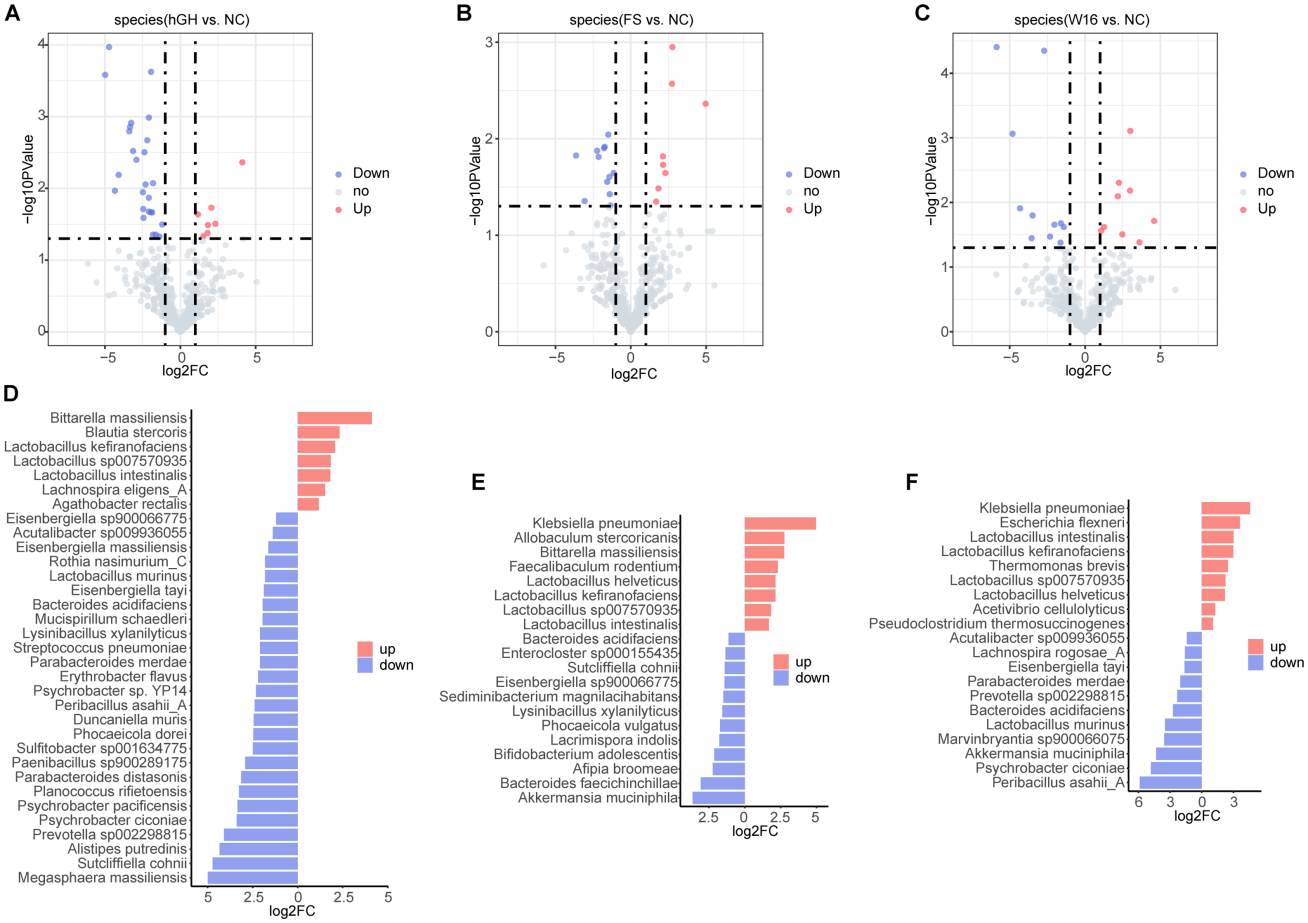
Differences in the distribution of gut microbiota in different treatment groups. **(A)** Volcano of differential bacteria in hGH group vs. NC group. **(B)** Volcano of differential bacteria in FS group vs. NC group. **(C)** Volcano of differential bacteria in W16 group vs. NC group. **(D)** Bidirectional bar chart shows the specific differential species in hGH group vs. NC group. **(E)** Bidirectional bar chart shows the specific differential species in FS group vs. NC group. **(F)** Bidirectional bar chart shows the specific differential species in W16 group vs. NC group.

Next, we conducted non-parametric testing analysis to identify species enriched or downregulated by different treatments. In comparison with NC group, hGH, unfermented and fermented *Astragali Radix* treatment upregulated 7, 8, and 9 species, respectively, while downregulating 26, 12, and 11 species (Figures 6A–C). Specifically, hGH boosted populations of *Bittarella massiliensis*, *Blautia stercoris*, *Lactobacillus kefiranofaciens*, *Lactobacillus sp007570935*, *Lactobacillus intestinalis*, *Lachnospira eligens_A*, while decreasing *Megasphaera massiliensis*, *Sutcliffiella cohnii*, *Alistipes putredinis*, *Prevotella sp002298815*, *Psychrobacter ciconiae*, et al. (Figure 6D). Unfermented *Astragali Radix* treatment prominently elevated the presence of *Klebsiella pneumoniae*, *Allobaculum stercoricanis*, *Bittarella massiliensis*, *Faecalibaculum rodentium*, *Lactobacillus helveticus*, *Lactobacillus kefiranofaciens*, *Lactobacillus sp007570935*, while diminished *Akkermansia muciniphila*, *Bacteroides faecichinchillae*, *Afipia broomeae*, *Bifidobacterium adolescentis*, *Lacrimispora indolis*, *Phocaeicola vulgatus*, *Lysinibacillus xylanilyticus*, et al. (Figure 6E). Fermented *Astragali Radix* exposure favored species like *Klebsiella pneumoniae*, *Escherichia flexneri*, *Lactobacillus intestinalis*, *Lactobacillus kefiranofaciens*, *Lactobacillus sp007570935*, *Thermomonas brevis*, *Lactobacillus helveticus*, *Acetivibrio cellulolyticus*, *Pseudoclostridium thermosuccinogenes*, but suppressed *Peribacillus asahii_A*, *Psychrobacter ciconiae*, *Akkermansia muciniphila*, *Marvinbryantia sp900066075*, *Lactobacillus murinus*, *Bacteroides acidifaciens*, *Prevotella sp002298815*, *Parabacteroides merdae*, *Lachnospira rogosae_A*, *Acutalibacter sp009936055* (Figure 6F).

### 3.6 Fermented *Astragali Radix* alters the metabolism of gut microbiota community

**Figure 7:**
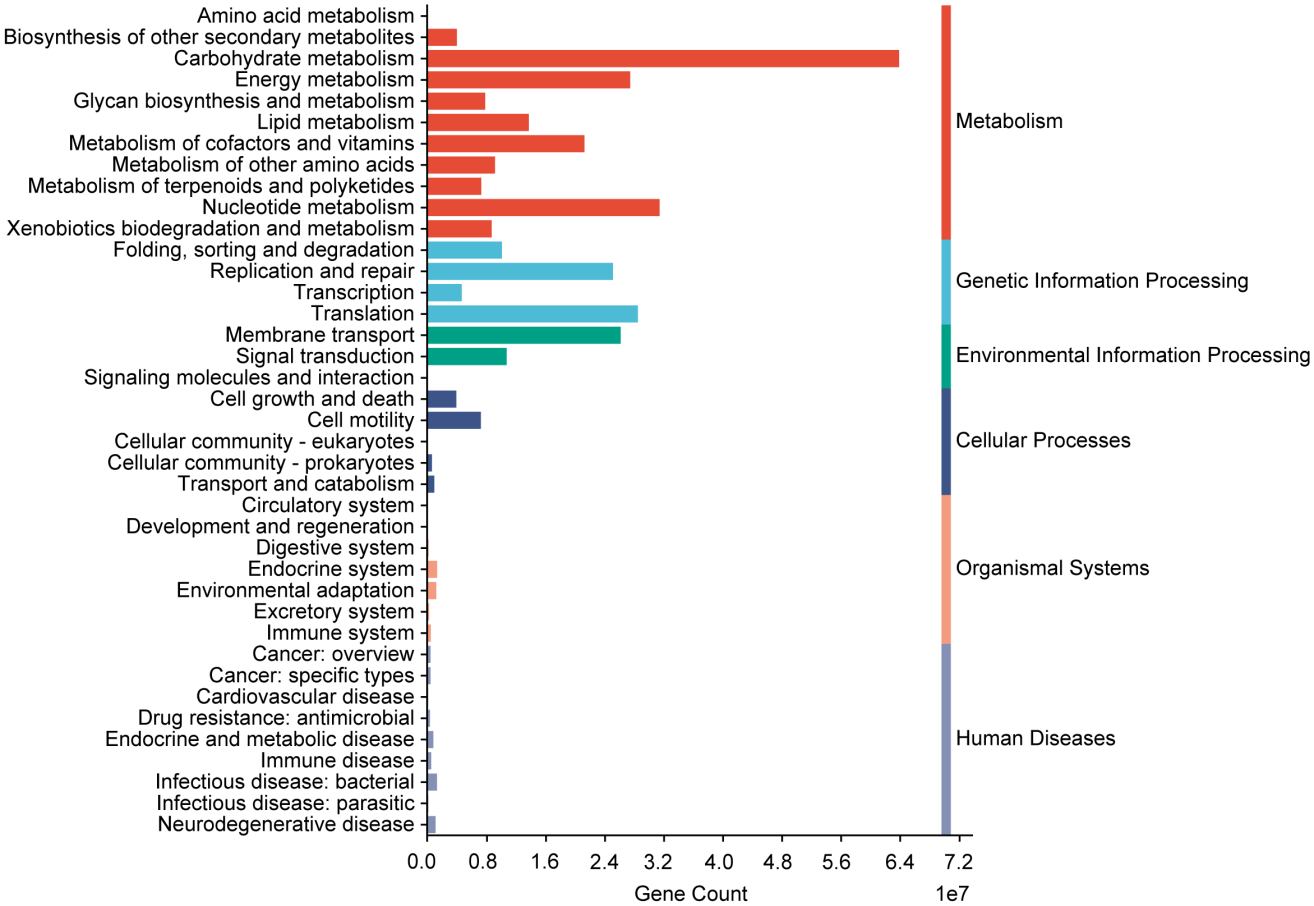
The function of microbial genes predicted by PICRUSt2 based on annotations from KEGG databases.

Compelling studies have proved that human metabolism disorders, including abnormalities in glucose, amino acid, lipid and energy metabolism, can disrupt the balance of bone homeostasis (Talarico et al., 2023). To investigate this, we utilized PICRUSt2 to predict the functional capacities of the gut microbiota. The KEGG pathway annotations revealed that the identified pathways were predominantly related to metabolism, encompassing amino acid metabolism, carbohydrate metabolism, energy metabolism, lipid metabolism, and glycan biosynthesis and metabolism (Figure 7). Based on these findings, we hypothesize that *Bifidobacterium animalis* subsp. *lactis* BL-16 fermented *Astragali Radix* may enhance bone development by modulating gut microbiota and improving metabolic processes.

### 3.7 Component analysis of *Astragali Radix* before and after fermentation

To investigate the material basis for the osteogenic activity of FS and W16, we conducted component detection and identification using UPLC-Q-TOF/MS (Supplementary Figures 1A,B). In this study, a total of 85 metabolites were identified before fermentation and 113 metabolites after fermentation (Tables 1, 2). The fermentation process significantly remodeled the metabolite composition of the samples, manifested by a notable increase in the variety and chemical diversity of metabolites. The metabolites that specifically accumulated after fermentation were mainly enriched in flavonoids (such as genistein, baicalein, chrysoeriol, etc.), phenolic acids (such as ferulic acid, sinapyl alcohol, etc.), and lipid metabolites (such as phytosphingosine, linolenic acid, etc.). This suggests that fermentation may enhance the biological activity of the product by activating the phenylpropanoid metabolic pathway, promoting the release of cell wall-derived compounds, and regulating membrane lipid remodeling. In contrast, the metabolites unique to the pre-fermentation stage were mostly sugars (such as stachyose, maltotriose) and early intermediate metabolites (such as glycerophosphocholine). It is speculated that these were preferentially utilized by microorganisms as carbon sources or energy substrates during the fermentation process. Common metabolites such as choline, gluconic acid, proline, and ursolic acid were stably present both before and after fermentation, indicating their sustained role in basic metabolic regulation. Overall, fermentation not only enriched the chemical structural types of metabolites but also induced the generation of various secondary metabolites with potential functional activities, providing an important material basis for subsequent research into functional mechanisms.

**Table 1:** Profiling of FS extract via UPLC-Q-TOF/MS.

No.	Name	RT (min)	Mass	Theoretical mass	Formula	Ionization model
1	Acetylenedicarboxylic acid	0.717	112.98511	112.98803	C_4_H_2_O_4_	ESI^−^
2	Choline	0.753	104.10759	104.10699	C_5_H_14_NO	ESI^+^
3	L-Asparagine	0.767	131.04558	131.04622	C_4_H_8_N_2_O_3_	ESI^−^
4	L-Carnitine	0.774	162.11264	162.11247	C_7_H_15_NO_3_	ESI^+^
5	Taurine	0.789	124.00684	124.00739	C_2_H_7_NO_3_S	ESI^−^
6	D-Aspartate	0.789	132.02975	132.03023	C_4_H_7_NO_4_	ESI^−^
7	N-Acetyl-DL-Serine	0.796	146.04565	146.04588	C_5_H_9_NO_4_	ESI^−^
8	Glycerophosphocholine	0.796	258.11026	258.11011	C_8_H_20_NO_6_P	ESI^+^
9	Maltotriose	0.803	539.14001	539.13733	C_18_H_32_O_16_	ESI^−^
10	Stachyose	0.803	665.21411	665.2146	C_24_H_42_O_21_	ESI^−^
11	Galactitol	0.803	181.071	181.07176	C_6_H_14_O_6_	ESI^−^
12	L-Glutamic acid	0.803	148.06082	148.06044	C_5_H_9_NO_4_	ESI^+^
13	Fructose	0.81	215.0314	215.03169	C_6_H_12_O_6_	ESI^−^
14	Maltose	0.824	341.10873	341.10895	C_12_H_22_O_11_	ESI^−^
15	Gluconic acid	0.824	195.05008	195.05103	C_6_H_12_O_7_	ESI^−^
16	Phloroglucinol	0.824	127.0387	127.03897	C_6_H_6_O_3_	ESI^+^
17	2-(Hydroxymethyl)-3-methoxy-2H-furan-5-one	0.824	145.04985	145.04953	C_6_H_8_O_4_	ESI^+^
18	Disaccharide	0.824	325.11377	325.11292	C_12_H_22_O_11_	ESI^+^
19	Sucrose	0.832	360.15036	360.15002	C_12_H_22_O_11_	ESI^+^
20	L-Proline	0.832	116.07132	116.07061	C_5_H_9_NO_2_	ESI^+^
21	Succinic acid	0.881	119.03608	119.03388	C_4_H_6_O_4_	ESI^+^
22	Adenine	1.009	136.06158	136.06177	C_5_H_5_N_5_	ESI^+^
23	Xanthine	1.222	153.04111	153.0407	C_5_H_4_N_4_O_2_	ESI^+^
24	2-Hydroxy-6-methylisonicotinic acid	8.11	154.0509	154.05	C_7_H_7_NO_3_	ESI^+^
25	4-Hydroxybenzaldehyde	8.957	121.02889	121.0295	C_7_H_6_O_2_	ESI^−^
26	7-Hydroxy-coumarin	9.356	161.02469	161.02441	C_9_H_6_O_3_	ESI^−^
27	Cinnamaldehyde	10.259	133.065	133.06479	C_9_H_8_O	ESI^+^
28	Trans-p-hydroxycinnamic acid	10.266	163.0396	163.04007	C_9_H_8_O_3_	ESI^−^
29	4H-Chromen-4-one	10.266	147.04388	147.04401	C_9_H_6_O_2_	ESI^+^
30	4′-Methylgenistein	10.594	283.06067	283.06119	C_16_H_12_O_5_	ESI^−^
31	Glyceric acid	10.594	211.03976	211.04594	C_3_H_6_O_4_	ESI^−^
32	Beta-Sitosterol	10.607	453.34366	453.3493	C_29_H_50_O	ESI^+^
33	Ferulate	10.843	193.05074	193.05063	C_10_H_10_O_4_	ESI^−^
34	Ferulic acid	10.85	195.06606	195.06519	C_10_H_10_O_4_	ESI^+^
35	Sinapinic acid	10.914	223.06422	223.0612	C_11_H_12_O_5_	ESI^−^
36	Hesperedin	10.942	609.18481	609.1825	C_28_H_34_O_15_	ESI^−^
37	Isovitexin	11.098	433.11316	433.11301	C_21_H_20_O_10_	ESI^+^
38	Chrysoeriol (Luteolin 3′-methyl ether)	11.327	299.05676	299.05612	C_16_H_12_O_6_	ESI^−^
39	3-Isobutylglutaric acid	11.419	187.09787	187.09758	C_9_H_16_O_4_	ESI^−^
40	Swertisin	11.448	445.11426	445.11401	C_22_H_22_O_10_	ESI^−^
41	Apigetrin	11.618	431.09818	431.09836	C_21_H_20_O_10_	ESI^−^
42	Isoeugenitol	11.625	207.06512	207.06519	C_11_H_10_O_4_	ESI^+^
43	Phloretin	11.767	275.09229	275.0914	C_15_H_14_O_5_	ESI^+^
44	Isosakuranetin	11.775	285.07709	285.07684	C_16_H_14_O_5_	ESI^−^
45	Biotin	11.945	283.0509	283.0513	C_10_H_16_N_2_O_3_S	ESI^+^
46	Sinapyl alcohol	11.946	209.0791	209.08194	C_11_H_14_O_4_	ESI^−^
47	Nonanoicacid_major	11.952	211.0938	211.09409	C_9_H_16_O_4_	ESI^+^
48	4-Hydroxybenzoate	12.003	137.0242	137.02441	C_7_H_6_O_3_	ESI^−^
49	Xanthone	12.522	197.05984	197.05971	C_13_H_8_O_2_	ESI^+^
50	Ononin	12.522	431.13458	431.134	C_22_H_22_O_9_	ESI^+^
51	6-Hydroxy-2-(4-methoxyphenyl)-4H-chromen-4-one	12.529	269.08163	269.082	C_16_H_12_O_4_	ESI^+^
52	(−)-12-Hydroxyjasmonic acid	12.749	225.11378	225.11324	C_12_H_18_O_4_	ESI^−^
53	Baicalein	13.091	269.04526	269.04553	C_15_H_10_O_5_	ESI^−^
54	Formononetine	13.091	269.08081	269.08084	C_16_H_12_O_4_	ESI^+^
55	Methylnissolin-3-O-glucoside	13.091	463.16031	463.16	C_23_H_26_O_10_	ESI^+^
56	Isomer of dihydrophaseic acid	13.112	283.15497	283.15399	C_15_H_22_O_5_	ESI^+^
57	Benzoic acid	13.133	123.04446	123.04405	C_7_H_6_O_2_	ESI^+^
58	Butein	13.312	271.05994	271.06119	C_15_H_12_O_5_	ESI^−^
59	Cinnamic acid	13.319	149.05992	149.06	C_9_H_8_O_2_	ESI^+^
60	Glucosamine 6-phosphate	13.319	260.05176	260.05298	C_6_H_14_NO_8_P	ESI^+^
61	Kumatakenin	13.874	315.08688	315.08701	C_17_H_14_O_6_	ESI^+^
62	Jasmonic acid	14.528	211.13336	211.13287	C_12_H_18_O_3_	ESI^+^
63	9-Methoxycarbonyldec-9-enoic acid	15.418	227.12901	227.12888	C_12_H_20_O_4_	ESI^−^
64	10-Hydroxydecanoate	15.852	187.13382	187.13397	C_10_H_20_O_3_	ESI^−^
65	Ursolic acid	15.873	457.36771	457.36761	C_30_H_48_O_3_	ESI^+^
66	Methyl jasmonic acid	16.634	223.13414	223.13397	C_13_H_20_O_3_	ESI^−^
67	Isopalmitic acid	16.94	274.27554	274.27405	C_16_H_32_O_2_	ESI^+^
68	8-Hydroxy-6,7-dimethoxy-2H-chromen-2-one	17.154	223.06367	223.063	C_11_H_10_O_5_	ESI^+^
69	Isoastragaloside II	17.175	849.46014	849.46002	C_43_H_70_O_15_	ESI^+^
70	Astragaloside Iv_major	18.207	807.45093	807.45013	C_41_H_68_O_14_	ESI^+^
71	13-OxoODE	19.288	295.22598	295.22678	C_18_H_30_O_3_	ESI^+^
72	Linolenic acid	22.904	279.23196	279.23184	C_18_H_30_O_2_	ESI^+^
73	2-Phenylethyl b-D-glucopyranoside	24.355	307.1192	307.12	C_14_H_20_O_6_	ESI^+^
74	Palmitoleic acid	27.243	253.21678	253.2173	C_16_H_30_O_2_	ESI^−^
75	Stearic acid	27.349	283.26401	283.26425	C_18_H_36_O_2_	ESI^−^
76	Elaidic acid	27.349	281.24875	281.2486	C_18_H_34_O_2_	ESI^−^
77	Eicosenoic acid	27.371	309.28116	309.27991	C_20_H_38_O_2_	ESI^−^
78	Arachidic acid	27.378	311.2955	311.29556	C_20_H_40_O_2_	ESI^−^
79	Linoleic acid	27.542	279.23288	279.23294	C_18_H_32_O_2_	ESI^−^
80	Octadecanedioic acid	27.72	315.25269	315.25299	C_18_H_34_O_4_	ESI^+^
81	Guanidoacetic acid	27.748	233.10426	233.10037	C_3_H_7_N_3_O_2_	ESI^−^
82	6-Amino-9H-purine-9-propanoic acid	27.749	208.08304	208.0829	C_8_H_9_N_5_O_2_	ESI^+^
83	10-Heptadecenoic acid	28.032	267.23257	267.23294	C_17_H_32_O_2_	ESI^−^
84	(R,R)-Tartaric acid	28.345	149.0101	149.00916	C_4_H_6_O_6_	ESI^−^
85	1,4-Cyclohexanedicarboxylic acid	29.143	173.07912	173.08084	C_8_H_12_O_4_	ESI^+^

**Table 2:** Profiling of W16 extract via UPLC-Q-TOF/MS.

No.	Name	RT (min)	Mass	Theoretical mass	Formula	Ionization model
1	Canavanine	0.71	175.08397	175.08366	C_5_H_12_N_4_O_3_	ESI^−^
2	L-Arginine	0.71	175.12027	175.11896	C_6_H_14_N_4_O_2_	ESI^+^
3	Choline	0.753	104.10767	104.10645	C_5_H_14_NO	ESI^+^
4	Gluconic acid	0.824	195.04958	195.05103	C_6_H_12_O_7_	ESI^−^
5	Squamatic acid	0.824	411.07202	411.06976	C_19_H_18_O_9_	ESI^−^
6	Maltose	0.824	341.10773	341.10895	C_12_H_22_O_11_	ESI^−^
7	2-(Hydroxymethyl)-3-methoxy-2H-furan-5-one	0.824	145.0509	145.04953	C_6_H_8_O_4_	ESI^+^
8	Trigonelline	0.824	138.05606	138.05496	C_7_H_7_NO_2_	ESI^+^
9	Thamnolic acid	0.832	457.0206	457.01785	C_19_H_16_O_11_	ESI^−^
10	Proline	0.832	116.07172	116.0706	C_5_H_9_NO_2_	ESI^+^
11	Indole-3-acetaldehyde	0.895	158.05566	158.06114	C_10_H_9_NO	ESI^−^
12	N-Acetylindole	0.895	160.07268	160.07568	C_10_H_9_NO	ESI^+^
13	2-Aminophenol	0.924	110.06075	110.06004	C_6_H_7_NO	ESI^+^
14	L-Pipecolic acid	0.924	130.08833	130.08626	C_6_H_11_NO_2_	ESI^+^
15	3-Methyladenine	0.938	150.0788	150.07742	C_6_H_7_N_5_	ESI^+^
16	1-Hydroxy-2-naphthoic acid	1.173	187.04218	187.04007	C_11_H_8_O_3_	ESI^−^
17	Nicotinic acid	1.173	124.0405	124.03931	C_6_H_5_NO_2_	ESI^+^
18	Mandelic acid	1.173	205.02888	205.02614	C_9_H_10_O_3_	ESI^+^
19	5-Oxo-D-proline	1.244	128.03452	128.03532	C_5_H_7_NO_3_	ESI^−^
20	N-Acetyl-DL-glutamic acid	1.323	188.05855	188.05644	C_7_H_11_NO_5_	ESI^−^
21	Propionic acid	1.515	73.02912	73.0295	C_3_H_6_O_2_	ESI^−^
22	3-Hydroxy-3-methylglutaric acid	1.664	161.04402	161.04555	C_6_H_10_O_5_	ESI^−^
23	4-Methyl-5-thiazoleethanol	2.112	144.0484	144.04776	C_6_H_9_NOS	ESI^+^
24	Pyridoxamine	2.639	169.09818	169.09715	C_8_H_12_N_2_O_2_	ESI^+^
25	2-Isopropylmalic acid	7.421	175.06128	175.0612	C_7_H_12_O_5_	ESI^−^
26	4-Hydroxybenzoate	7.478	137.02441	137.02441	C_7_H_6_O_3_	ESI^−^
27	2-(1-Hydroxyethyl)-4-(2-hydroxypropyl)-2H-furan-5-one	7.94	185.08253	185.08194	C_9_H_14_O_4_	ESI^−^
28	Mycophenolic acid	8.438	319.11792	319.11871	C_17_H_20_O_6_	ESI^−^
29	6-Carboxyhexanoate	8.616	159.06525	159.06628	C_7_H_12_O_4_	ESI^−^
30	2-Hydroxyisocaproic acid	8.816	131.07051	131.07137	C_6_H_12_O_3_	ESI^−^
31	Divaric acid	8.915	195.06558	195.06628	C_10_H_12_O_4_	ESI^−^
32	Asaraldehyde	8.921	197.08157	197.08084	C_10_H_12_O_4_	ESI^+^
33	N-Acetyl-L-Leucine	9.349	172.09732	172.09792	C_8_H_15_NO_3_	ESI^−^
34	7-Hydroxy-coumarin	9.355	163.04018	163.03897	C_9_H_6_O_3_	ESI^+^
35	2-[(2-Hydroxy-3-methylbutanoyl)amino]-4-methylpentanoic acid	9.819	230.13945	230.13979	C_11_H_21_NO_4_	ESI^−^
36	(−)-12-Hydroxyjasmonic acid	9.911	225.11276	225.11324	C_12_H_18_O_4_	ESI^−^
37	Methyl haematommate	10.025	209.04506	209.04555	C_10_H_10_O_5_	ESI^−^
38	Quercitrin	10.096	447.09399	447.09329	C_21_H_20_O_11_	ESI^−^
39	Trans-cinnamate	10.152	131.04915	131.04913	C_9_H_8_O_2_	ESI^+^
40	6-Methylcoumarin	10.266	161.05956	161.05971	C_10_H_8_O_2_	ESI^+^
41	Trans-cinnamic acid	10.274	147.04443	147.04515	C_9_H_8_O_2_	ESI^−^
42	N-Acetyl-O-methyltyrosine	10.452	236.0925	236.09283	C_12_H_15_NO_4_	ESI^−^
43	2-Hydroxyoctanoic acid	10.573	159.10242	159.10266	C_8_H_16_O_3_	ESI^−^
44	2,4,5-Trimethoxybenzoic acid	10.593	251.02664	251.03163	C_10_H_12_O_5_	ESI^+^
45	4-Methoxy-7-methyl-5H-furo[3,2-g]chromen-5-one	10.593	253.04932	253.05	C_13_H_10_O_4_	ESI^+^
46	Vanillin	10.594	151.03981	151.04007	C_8_H_8_O_3_	ESI^−^
47	Velutin	10.736	313.07236	313.07175	C_17_H_14_O_6_	ESI^−^
48	3-O-Acetylpinobanksin	10.742	315.08597	315.0863	C_17_H_14_O_6_	ESI^+^
49	Tryptophan	10.95	203.08006	203.0826	C_11_H_12_N_2_O_2_	ESI^−^
50	3-Oxo-3-[[(2R,3S,4S,5R,6S)-3,4,5-trihydroxy-6-(4-hydroxy-5-methyl-2-propan-2-ylphenoxy)oxan-2-yl]methoxy]propanoic acid	10.964	413.14642	413.14532	C_19_H_26_O_10_	ESI^−^
51	N-Cinnamoylglycine	11.029	204.06587	204.06662	C_11_H_11_NO_3_	ESI^−^
52	Apigetrin	11.105	433.11383	433.11292	C_21_H_20_O_10_	ESI^+^
53	Genistein	11.105	271.05548	271.06009	C_15_H_10_O_5_	ESI^+^
54	[1-(7-Methoxy-2-oxochromen-8-yl)-3-methyl-2-oxobutyl] acetate	11.185	317.10461	317.10306	C_17_H_18_O_6_	ESI^−^
55	(4-Oxido-2,3,5,6,7,8-hexahydro-1H-pyrrolizin-4-ium-1-yl)methyl 2,3-dihydroxy-3-methylpentanoate	11.249	286.16638	286.16599	C_14_H_25_NO_5_	ESI^−^
56	3-Isobutylglutaric acid	11.419	187.09727	187.09758	C_9_H_16_O_4_	ESI^−^
57	Indole-3-carboxyaldehyde	11.519	144.04527	144.04549	C_9_H_7_NO	ESI^−^
58	5-(Hydroxymethyl)-3-(1-hydroxy-5-methylhexyl)oxolan-2-one_major	11.54	231.16306	231.15909	C_12_H_22_O_4_	ESI^+^
59	Sinapinic acid	11.633	223.06119	223.0612	C_11_H_12_O_5_	ESI^−^
60	4-Hydroxy-3,3,5-trimethyl-4-[(E)-3-[3,4,5-trihydroxy-6-(hydroxymethyl)oxan-2-yl]oxybut-1-enyl]cyclohexan-1-one	11.661	406.24835	406.24353	C_19_H_32_O_8_	ESI^+^
61	Chrysoeriol	11.697	299.05542	299.05612	C_16_H_12_O_6_	ESI^−^
62	3,16,17-Trihydroxy-17-acetyl-androstane	11.739	351.2562	351.25299	C_21_H_34_O_4_	ESI^+^
63	2,4-Dihydroxyheptadec-16-enyl acetate [IIN-based on: CCMSLIB00000848387]	11.739	679.51184	679.51196	C_19_H_36_O_4_	ESI^+^
64	(5R)-5-Hydroxy-1-(4-hydroxy-3-methoxyphenyl)tetradecan-3-one [IIN-based on: CCMSLIB00000848859]	11.739	701.49329	701.49872	C_21_H_34_O_4_	ESI^+^
65	Sinapyl alcohol	11.946	209.07913	209.08194	C_11_H_14_O_4_	ESI^−^
66	(2R,3S,4S,5R,6S)-2-(Hydroxymethyl)-6-[4-hydroxy-2-(3-methylbut-2-enyl)phenoxy]oxane-3,4,5-triol	11.946	399.16223	399.16605	C_17_H_24_O_7_	ESI^−^
67	Azelaic_acid	11.946	397.18542	397.18439	C_9_H_16_O_4_	ESI^−^
68	Baicalein	11.946	269.04544	269.04553	C_15_H_10_O_5_	ESI^−^
69	Ciprofibrate	11.946	287.02191	287.02472	C_13_H_14_Cl_2_O_3_	ESI^−^
70	Delphinidin-3-glucoside	11.946	464.09207	464.09604	C_21_H_21_O_12_	ESI^−^
71	6-Amino-9H-purine-9-propanoic acid	11.952	208.08583	208.0829	C_8_H_9_N_5_O_2_	ESI^+^
72	Biotin	11.952	283.05115	283.0513	C_10_H_16_N_2_O_3_S	ESI^+^
73	(5E)-3,4,9-Trihydroxy-2-propyl-2,3,4,7,8,9-hexahydrooxecin-10-one	12.032	243.12334	243.12379	C_12_H_20_O_5_	ESI^−^
74	(E)-5-(4-Methoxy-5-methyl-6-oxopyran-2-yl)-3-methylhex-4-enoic acid	12.032	265.1055	265.10815	C_14_H_18_O_5_	ESI^−^
75	MMV688372	12.145	400.15549	400.1579	C_23_H_20_FN_5_O	ESI^−^
76	Simonyellin	12.145	273.04324	273.04047	C_14_H_10_O_6_	ESI^−^
77	(2R)-2-[(2R,5S)-5-[(2S)-2-Hydroxybutyl]oxolan-2-yl]propanoic acid	12.294	199.133	199.13287	C_11_H_20_O_4_	ESI^+^
78	1-(1-Hydroxybutyl)-1,3,4,5,6,7-hexahydro-2-benzofuran-4,5,6,7-tetrol	12.316	259.11841	259.11871	C_12_H_20_O_6_	ESI^−^
79	Ononin	12.523	429.12134	429.11911	C_22_H_22_O_9_	ESI^−^
80	Kuhlmannin	12.615	297.07767	297.07684	C_17_H_14_O_5_	ESI^−^
81	S-(5’-Adenosyl)-L-homocysteine	12.978	383.11426	383.11432	C_14_H_20_N_6_O_5_S	ESI^−^
82	Chrysin	13.091	253.04956	253.05063	C_15_H_10_O_4_	ESI^−^
83	3,4-Dimethoxybenzaldehyde	13.112	167.07063	167.07027	C_9_H_10_O_3_	ESI^+^
84	1-(2,4-Dihydroxyphenyl)-2-(3,5-dimethoxyphenyl)propan-1-one	13.319	301.10764	301.10815	C_17_H_18_O_5_	ESI^−^
85	Benzoic acid	13.319	123.0444	123.04405	C_7_H_6_O_2_	ESI^+^
86	Rel-dimethylenedioxy-dimethoxy-epoxylignan	13.596	401.15915	401.15948	C_22_H_24_O_7_	ESI^+^
87	4′-Methylgenistein (biochanin A)	13.617	307.05234	307.05771	C_16_H_12_O_5_	ESI^+^
88	Glyceric acid	13.618	211.03966	211.04594	C_3_H_6_O_4_	ESI^−^
89	9-Methoxycarbonyldec-9-enoic acid	14.529	227.12823	227.12888	C_12_H_20_O_4_	ESI^−^
90	Eriodictyol 7,3′-dimethyl ether	14.948	315.08624	315.0874	C_17_H_16_O_6_	ESI^−^
91	Dihydrojasmonic acid	15.454	211.1329	211.13397	C_12_H_20_O_3_	ESI^−^
92	[5-Acetyloxy-3-(hydroxymethyl)-2-oxo-6-propan-2-ylcyclohex-3-en-1-yl] 3-methylpentanoate	15.788	339.18213	339.1813	C_18_H_28_O_6_	ESI^−^
93	Ursolic acid	15.866	439.3555	439.35706	C_30_H_48_O_3_	ESI^+^
94	7-Methoxy-4-methylcoumarin	16.798	191.07066	191.07027	C_11_H_10_O_3_	ESI^+^
95	(2S,3R,4S,5R)-2-[(2R,3R,4S,5S,6R)-4,5-Dihydroxy-6-(hydroxymethyl)-2-(2-phenylethoxy)oxan-3-yl]oxyoxane-3,4,5-triol	16.941	415.16229	415.16098	C_19_H_28_O_10_	ESI^−^
96	Phytosphingosine	17.125	318.30121	318.30026	C_18_H_39_NO_3_	ESI^+^
97	Ribitol	17.175	175.06093	175.05769	C_5_H_12_O_5_	ESI^+^
98	Isoastragaloside II	18.008	849.46149	849.46002	C_43_H_70_O_15_	ESI^+^
99	3-Hydroxy-5,5,8a-trimethyl-3,4,4a,6,7,8-hexahydronaphthalene-2-carboxylic acid	18.2	237.15001	237.14961	C_14_H_22_O_3_	ESI^−^
100	Echinocystic acid-3-O-glucoside	19.431	633.40375	633.40082	C_36_H_58_O_9_	ESI^−^
101	(6E)-2,6,10-Trimethyldodeca-6,11-diene-2,3,10-triol_major	22.547	257.21277	257.21112	C_15_H_28_O_3_	ESI^+^
102	(4S,5Z,6S)-4-(2-Methoxy-2-oxoethyl)-5-[2-[(E)-3-phenylprop-2-enoyl]oxyethylidene]-6-[(2S,3R,4S,5S,6R)-3,4,5-trihydroxy-6-(hydroxymethyl)oxan-2-yl]oxy-4H-pyran-3-carboxylic acid	23.031	233.15312	233.15471	C_15_H_22_O_2_	ESI^−^
103	Planchol A	24.347	279.08237	279.0863	C_14_H_14_O_6_	ESI^+^
104	4-Hydroxybenzoic acid	24.354	139.03996	139.03897	C_7_H_6_O_3_	ESI^+^
105	5-[(2R,3R,4S,5R,6R)-3,5-Dihydroxy-2-(hydroxymethyl)-6-(2-phenylethoxy)oxan-4-yl]oxy-3-hydroxy-3-methyl-5-oxopentanoic acid	24.354	446.19592	446.20209	C_20_H_28_O_10_	ESI^+^
106	Excavatin L	24.354	381.08557	381.09	C_19_H_18_O_7_	ESI^+^
107	Lipoic acid, reduced	24.354	209.0706	209.06645	C_8_H_16_O_2_S_2_	ESI^+^

## 4 Discussion

Short stature, characterized by abnormal growth in children, affects approximately 3% of the individuals (Wang et al., 2025). The pathogenesis of short stature involves multiple factors, and its exact etiology remains unclear. It is well-established that the colonization of the infant gut microbiota begins at birth and matures as the child grows. Therefore, the gut microbiota plays a key role in bone growth (Guo et al., 2022). Moreover, *Astragali Radix* has been reported to positively influence bone growth and development (Lee et al., 2017). This study is conducted to validate the growth-promoting effects of *Astragali Radix*, as well as compare them with those of probiotic fermented *Astragali Radix*. Our findings indicate that fermented *Astragali Radix* outperforms its unfermented counterpart. These results offer a promising approach for addressing short stature in children.

Human growth hormone (hGH) is the guideline-recommended therapy for short stature. Research has consistently shown that hGH effectively increases the growth rate and final height of children with short stature (Kim et al., 2014). However, clinical evidence indicates that hGH injections can lead to adverse reactions, such as elevated blood glucose levels and insulin resistance (Kim et al., 2021). In our presented study, hGH was found to promote bone growth in juvenile rats, but it also resulted in increased blood glucose and insulin levels. Staggeringly, both unfermented and fermented *Astragali Radix* not only enhanced growth in juvenile rats but also did not increase glucose or insulin levels. Notably, the effects were more pronounced with *Bifidobacterium animalis* subsp. *lactis* BL-16 fermented *Astragali Radix*. These results suggest that probiotic-fermented *Astragali Radix* may offer a superior alternative for promoting bone growth without the negative metabolic side effects associated with hGH therapy.

It is unquestionable that an indispensable role of gut microbiota in host physiological functions and over health (Zhong et al., 2024; Xu et al., 2023; Dong et al., 2023; Yang Z. et al., 2023). Disruption of gut microbiota can impair intestinal immunity, inhibit intestinal calcium uptake and affect osteoclast-mediated bone resorption, all of which impact the bone mass (Liu et al., 2023). To explore the potential species involved in the bone-promoting effects of *Astragali Radix*, we analyzed changes in the gut microbial community following different treatments. Notably, treatment with hGH, unfermented and fermented *Astragali Radix* all boosted the growth of *Lactobacillus* species (*L. kefiranofaciens*, *L. sp00757093*5, *L. helveticus*, *L. intestinalis*). Existing evidences have proved that these species can upregulate the expression of genes such as TRPV 5, TRPV 6, PepT 1, Calbindin-D9k, and the calcium pump, leading to enhanced calcium absorption rate and increased bone mineral density (Hu et al., 2024; Lim et al., 2021). Additionally, Chen et al. (2017) reported that postmenopausal women with normal bone mineral density had higher relative abundances of *Acetivibrio cellulolyticus* compared to those with osteoporosis. This suggests that the enrichment of *Acetivibrio cellulolyticus* through fermented *Astragali Radix* might contribute positively to bone growth.

The skeletal growth in childhood is regulated by complex interactions among various factors, with the GH/IGF-1 axis being the most prominent endocrine regulator of bone growth. Growth hormone (GH) plays a crucial role in bone metabolism by stimulating the secretion of IGF-1, which promotes bone growth and increases bone mineral content (Talarico et al., 2023). In our study, we observed elevated plasma and liver IGF-1 levels and upregulated *igf-1* gene expression in bone and liver tissues following treatment with hGH and fermented *Astragali Radix*. Additionally, neurotransmitters are known to influence bone growth and development. For instance, clinical studies have shown that dopamine supplementation can enhance GH secretion and accelerate growth in children with growth disorders (Huseman et al., 1986). Consistent with this, our findings revealed that treatment with fermented *Astragali Radix* significantly increased dopamine levels compared to hGH and unfermented *Astragali Radix*. These results suggest that fermented *Astragali Radix* primarily promotes bone development by activating the IGF-1 signaling axis and modulating neurotransmitter levels. However, this study primarily reveals associations and does not establish causality. Although we observed elevated IGF-1 levels and upregulated dopamine following treatment with fermented *Astragali Radix* combined with hGH, the direct causal relationship between these changes and skeletal development remains unclear. Future studies utilizing gene knockout models or specific inhibitor interventions are warranted to further validate the direct mechanisms through which the IGF-1 signaling axis and neurotransmitters mediate the effects of fermented *Astragali Radix* on bone growth.

The significant increase in the variety and quantity of metabolites after fermentation is closely associated with the microbial transformation of substrates and the release of bound-state compounds. Consistently, this study found that the metabolites specifically accumulated after fermentation were predominantly flavonoids and phenolic acids, which play a positive role in bone metabolism. Genistein, at physiological concentrations, has been shown to promote bone formation in rat and human bone marrow cells (Dai et al., 2013). Baicalein has been confirmed to facilitate osteoblast differentiation by activating pathways such as Wnt/β-catenin (Chen et al., 2017). Chrysoeriol not only promotes osteoblast differentiation but also protects these cells from oxidative stress damage and has demonstrated inhibitory effects on bone destruction in arthritis models (Wu et al., 2022). Notably, ferulic acid, a phenolic acid, possesses well-defined osteogenic effects, including promoting the osteogenic differentiation of bone marrow mesenchymal stem cells, enhancing alkaline phosphatase activity and mineralized nodule formation, and alleviating osteoporosis by regulating signaling pathways such as GSK-3β/Lrp-5/ERK (Zhou et al., 2021). These fermentation-enriched components collectively constitute the core material basis responsible for the superior osteogenic activity of W16. Although our UPLC-Q-TOF/MS analysis revealed significant metabolome remodeling in W16, with a notable increase in potentially bioactive flavonoids and phenolic acids, the current experimental design does not definitively delineate the origin of the observed osteogenic activity. It remains unclear whether the enhanced bioactivity is primarily attributed to the biotransformation of Astragalus-specific constituents or to the *de novo* synthesis of bioactive metabolites by the BL-16 strain itself during fermentation. In future studies, we need to include a blank control of BL-16 fermentation without Astragalus to confirm the source of the osteogenic activity.

## 5 Conclusion

In summary, our study confirmed the growth-promoting effects of fermented *Astragali Radix*, demonstrating that it is more effective than unfermented *Astragali Radix*. These findings contribute valuable data on probiotic-fermented traditional Chinese medicines (TCMs) and offer an improved therapeutic option for children with short stature.

## Data Availability

The raw data have been deposited in the NCBI repository (https://www.ncbi.nlm.nih.gov) under accession number PRJNA1445541.

## Footnotes

1
http://www.drive5.com/usearch/manual/uchime_algo.html

2
https://www.arb-silva.de/

## Supplementary material

SUPPLEMENTARY FIGURE 1 Profiling of FS and W16 extract via UPLC-Q-TOF/MS. The spectra of FS and W16 in **(A)** negative ion mode and **(B)** positive ion mode.

## References

[ref1] Chen L. J. Hu B. B. Shi X. L. Ren M. M. Yu W. B. Cen S. D. . (2017). Baicalein enhances the osteogenic differentiation of human periodontal ligament cells by activating the Wnt/β-catenin signaling pathway. Arch. Oral Biol. 78, 100–108. doi: 10.1016/j.archoralbio.2017.01.019, 28222387

[ref2] Chu X. D. Zhang Y. R. Lin Z. B. Zhao Z. Huangfu S. C. Qiu S. H. . (2021). A network pharmacology approach for investigating the multi-target mechanisms of Huangqi in the treatment of colorectal cancer. Transl. Cancer Res. 10, 681–693. doi: 10.21037/tcr-20-2596, 35116401 PMC8798599

[ref3] Dai J. Li Y. Zhou H. Chen J. Chen M. Xiao Z. (2013). Genistein promotion of osteogenic differentiation through BMP2/SMAD5/RUNX2 signaling. Int. J. Biol. Sci. 9, 1089–1098. doi: 10.7150/ijbs.7367, 24339730 PMC3858582

[ref4] Dong C. Yang Y. Wang Y. Hu X. Wang Q. Gao F. . (2023). Gut microbiota combined with metabolites reveals unique features of acute myocardial infarction patients different from stable coronary artery disease. J. Adv. Res. 46, 101–112. doi: 10.1016/j.jare.2022.06.008, 35750287 PMC10105070

[ref9002] Edgar RC. UPARSE: highly accurate OTU sequences from microbial amplicon reads. Nature methods. (2013) 10:996–8. 23955772 10.1038/nmeth.2604

[ref9001] Edgar RC Haas BJ Clemente JC Quince C Knight R. UCHIME improves sensitivity and speed of chimera detection. Bioinformatics (Oxford, England). (2011) 27:2194–200. 21700674 10.1093/bioinformatics/btr381 PMC3150044

[ref5] Farquharson C. Jefferies D. (2000). Chondrocytes and longitudinal bone growth: the development of tibial dyschondroplasia. Poult. Sci. 79, 994–1004. doi: 10.1093/ps/79.7.994, 10901201

[ref6] Guo X. Zhong K. Zhang J. Hui L. Zou L. Xue H. . (2022). Gut microbiota can affect bone quality by regulating serum estrogen levels. Am. J. Transl. Res. 14, 6043–6055, 36247294 PMC9556462

[ref7] Hor Y. Y. Ooi C. H. Lew L. C. Jaafar M. H. Lau A. S. Lee B. K. . (2021). The molecular mechanisms of probiotic strains in improving ageing bone and muscle of d-galactose-induced ageing rats. J. Appl. Microbiol. 130, 1307–1322. doi: 10.1111/jam.14776, 32638482

[ref8] Hu W. Pei Z. Xia A. Jiang Y. Yang B. Liu X. . (2024). *Lactobacillus helveticus*-derived whey-calcium chelate promotes calcium absorption and bone health of rats fed a low-calcium diet. Nutrients 16:1127. doi: 10.3390/nu16081127, 38674818 PMC11053418

[ref9] Huang J. Cui L. Lin H. Song M. Sun S. (2024). Effects of *Clostridium butyricum* on production performance and bone development of laying hens. Vet. Sci. 11:160. doi: 10.3390/vetsci11040160, 38668427 PMC11053732

[ref10] Huh J. E. Kim S. J. Kang J. W. Nam D. W. Choi D. Y. Park D. S. . (2015). The standardized BHH10 extract, a combination of *Astragalus membranaceus*, *Cinnamomum cassia*, and *Phellodendron amurense*, reverses bone mass and metabolism in a rat model of postmenopausal osteoporosis. Phytother. Res. 29, 30–39. doi: 10.1002/ptr.5218, 25230217 PMC4303985

[ref11] Huseman C. A. Hassing J. M. Sibilia M. G. (1986). Endogenous dopaminergic dysfunction: a novel form of human growth hormone deficiency and short stature. J. Clin. Endocrinol. Metab. 62, 484–490. doi: 10.1210/jcem-62-3-484, 3080463

[ref12] Jiang S. Qu X. Liu S. Wei J. Yi X. Liu Y. . (2023). Proteomic identification of plasma components in *Tachypleus tridentatus* and their effects on the longitudinal bone growth rate in rats. Mar. Drugs 21:111. doi: 10.3390/md21020111, 36827152 PMC9961754

[ref13] Kim S. H. Kim M. Yim J. Kim M. Jang D. H. (2021). Transient neonatal diabetes mellitus in SHORT syndrome: a case report. Front. Pediatr. 9:650920. doi: 10.3389/fped.2021.650920, 34249805 PMC8261148

[ref14] Kim B. Park M. J. (2009). The influence of weight and height status on psychological problems of elementary schoolchildren through child behavior checklist analysis. Yonsei Med. J. 50, 340–344. doi: 10.3349/ymj.2009.50.3.340, 19568594 PMC2703755

[ref15] Kim H. S. Yang S. W. Yoo H. W. Suh B. K. Ko C. W. Chung W. Y. . (2014). Efficacy of short-term growth hormone treatment in prepubertal children with idiopathic short stature. Yonsei Med. J. 55, 53–60. doi: 10.3349/ymj.2014.55.1.53, 24339287 PMC3874918

[ref16] Labarta J. I. de Arriba A. Ferrer M. Loranca M. Martos J. M. Rodríguez A. . (2020). Growth and metabolic effects of long-term recombinant human growth hormone (rhGH) treatment in short children born small for gestational age: GH-RAST study. J. Pediatr. Endocrinol. Metab. 33, 923–932. doi: 10.1515/jpem-2019-0438, 32623373

[ref17] Laugel-Haushalter V. Bär S. Schaefer E. Stoetzel C. Geoffroy V. Alembik Y. . (2019). A new SLC10A7 homozygous missense mutation responsible for a milder phenotype of skeletal dysplasia with amelogenesis imperfecta. Front. Genet. 10:504. doi: 10.3389/fgene.2019.00504, 31191616 PMC6546871

[ref18] Lee D. Lee S. H. Lee Y. H. Song J. Kim H. (2017). Astragalus extract mixture HT042 increases longitudinal bone growth rate by upregulating circulatory IGF-1 in rats. Evid. Based Complement. Alternat. Med. 2017:6935802. doi: 10.1155/2017/6935802, 28713437 PMC5496125

[ref19] Lim E. Y. Song E. J. Kim J. G. Jung S. Y. Lee S. Y. Shin H. S. . (2021). *Lactobacillus intestinalis* YT2 restores the gut microbiota and improves menopausal symptoms in ovariectomized rats. Benefic. Microbes 12, 503–516. doi: 10.3920/bm2020.0217, 34463192

[ref20] Liu F. Kong A. Fu P. Cao Q. Q. Tao K. S. Liu D. Y. . (2021). *Lactobacillus rhamnosus* JYLR-005 prevents thiram-induced tibial dyschondroplasia by enhancing bone-related growth performance in chickens. Probiotics Antimicrob. Proteins 13, 19–31. doi: 10.1007/s12602-020-09670-7, 32504282

[ref21] Liu T. Yu H. Wang S. Li H. Du X. He X. (2023). Chondroitin sulfate alleviates osteoporosis caused by calcium deficiency by regulating lipid metabolism. Nutr. Metab. 20:6. doi: 10.1186/s12986-023-00726-3, 36747190 PMC9901125

[ref22] Nilsson O. Baron J. (2004). Fundamental limits on longitudinal bone growth: growth plate senescence and epiphyseal fusion. Trends Endocrinol. Metab. 15, 370–374. doi: 10.1016/j.tem.2004.08.004, 15380808

[ref23] Oh J. Jeon S. B. Lee Y. Lee H. Kim J. Kwon B. R. . (2015). Fermented red ginseng extract inhibits cancer cell proliferation and viability. J. Med. Food 18, 421–428. doi: 10.1089/jmf.2014.3248, 25658580

[ref24] Qiao J. Y. Chen R. Wang M. J. Bai R. Cui X. J. Liu Y. . (2021). Perturbation of gut microbiota plays an important role in micro/nanoplastics-induced gut barrier dysfunction. Nanoscale 13, 8806–8816. doi: 10.1039/D1NR00038A, 33904557

[ref25] Schwarzer M. Makki K. Storelli G. Machuca-Gayet I. Srutkova D. Hermanova P. . (2016). *Lactobacillus plantarum* strain maintains growth of infant mice during chronic undernutrition. Science 351, 854–857. doi: 10.1126/science.aad8588, 26912894

[ref26] Su W. Yang Y. Zhao X. Cheng J. Li Y. Wu S. . (2024). Potential efficacy and mechanism of eight mild-natured and bitter-flavored TCMs based on gut microbiota: a review. Chin. Herb. Med. 16, 42–55. doi: 10.1016/j.chmed.2023.08.001, 38375054 PMC10874767

[ref27] Talarico V. Nicoletti A. Raiola G. (2023). Endocrine and metabolic disorders in adolescent and adult patients born small for gestational age. Acta Biomed. 94:e2023220. doi: 10.23750/abm.v94i6.15428, 38054664 PMC10734237

[ref28] Wang J. H. Bose S. Kim H. G. Han K. S. Kim H. (2015). Fermented *Rhizoma Atractylodis Macrocephalae* alleviates high fat diet-induced obesity in association with regulation of intestinal permeability and microbiota in rats. Sci. Rep. 5:8391. doi: 10.1038/srep08391, 25684573 PMC4329570

[ref29] Wang H. M. Wang X. Wu X. Yu K. Jia S. Jiang W. (2025). Effects of 24-week jumping exercise on bone mineral density and linear growth in children with short stature: a prospective controlled trial. BMC Sports Sci. Med. Rehabil. 17:334. doi: 10.1186/s13102-025-01376-z, 41233903 PMC12616931

[ref9003] Wang Q Garrity GM Tiedje JM Cole JR. Naive Bayesian classifier for rapid assignment of rRNA sequences into the new bacterial taxonomy. Applied and environmental microbiology. (2007) 73:5261–7. 17586664 10.1128/AEM.00062-07 PMC1950982

[ref30] Wu J. Y. Chen Y. J. Fu X. Q. Li J. K. Chou J. Y. Yin C. L. . (2022). Chrysoeriol suppresses hyperproliferation of rheumatoid arthritis fibroblast-like synoviocytes and inhibits JAK2/STAT3 signaling. BMC Complement. Med. Ther. 22:73. doi: 10.1186/s12906-022-03553-w, 35296317 PMC8928618

[ref31] Xu C. Ji G. E. (2013). Bioconversion of flavones during fermentation in milk containing *Scutellaria baicalensis* extract by *Lactobacillus brevis*. J. Microbiol. Biotechnol. 23, 1422–1427. doi: 10.4014/jmb.1305.05001, 23851266

[ref32] Xu W. Yu J. Yang Y. Li Z. Zhang Y. Zhang F. . (2023). Strain-level screening of human gut microbes identifies *Blautia producta* as a new anti-hyperlipidemic probiotic. Gut Microbes 15:2228045. doi: 10.1080/19490976.2023.2228045, 37408362 PMC10324434

[ref33] Yan J. Herzog J. W. Tsang K. Brennan C. A. Bower M. A. Garrett W. S. . (2016). Gut microbiota induce IGF-1 and promote bone formation and growth. Proc. Natl. Acad. Sci. U.S.A. 113, E7554–E7563. doi: 10.1073/pnas.1607235113, 27821775 PMC5127374

[ref34] Yang Y. Lu W. Zhang X. Wu C. (2022). Gut fungi differentially response to the antipyretic (heat-clearing) and diaphoretic (exterior-releasing) traditional Chinese medicines in *Coptis chinensis*-conditioned gut microbiota. Front. Pharmacol. 13:1032919. doi: 10.3389/fphar.2022.1032919, 36467054 PMC9716107

[ref35] Yang Z. Wang Q. Liu Y. Wang L. Ge Z. Li Z. . (2023). Gut microbiota and hypertension: association, mechanisms and treatment. Clin. Exp. Hypertens. 45:2195135. doi: 10.1080/10641963.2023.2195135, 36994745

[ref36] Yang Y. N. Zhan J. G. Cao Y. Wu C. M. (2024). From ancient wisdom to modern science: gut microbiota sheds light on property theory of traditional Chinese medicine. J. Integr. Med. 22, 413–444. doi: 10.1016/j.joim.2024.06.001, 38937158

[ref37] Yang Y. Zhao X. Xie Y. Wu C. (2023). Modulative effect of *Physalis alkekengi* on both gut bacterial and fungal micro-ecosystem. Chin. Herb. Med. 15, 564–573. doi: 10.1016/j.chmed.2023.02.003, 38094014 PMC10715876

[ref38] Zhong S. Sun Y. Q. Huo J. X. Xu W. Y. Yang Y. N. Yang J. B. . (2024). The gut microbiota-aromatic hydrocarbon receptor (AhR) axis mediates the anticolitic effect of polyphenol-rich extracts from *Sanghuangporus*. iMeta 3:e180. doi: 10.1002/imt2.180, 38882491 PMC11170970

[ref39] Zhou W. Chen B. Shang J. Li R. (2021). Ferulic acid attenuates osteoporosis induced by glucocorticoid through regulating the GSK-3β/Lrp-5/ERK signalling pathways. Physiol. Int. 108, 317–341. doi: 10.1556/2060.2021.00180, 34529586

[ref40] Zhou J. Cheng J. Liu L. Luo J. Peng X. (2023). *Lactobacillus acidophilus* (LA) fermenting Astragalus polysaccharides (APS) improves calcium absorption and osteoporosis by altering gut microbiota. Foods 12:275. doi: 10.3390/foods12020275, 36673366 PMC9858548

