# Supplementary figures and images for: Bifidobacterium animalis subsp. lactis BL-16 fermented Astragali Radix (W16) promotes the bone growth of juvenile rats via modulation of IGF-1 and gut microbiota
Source: Front Microbiol. 2026 Apr 14;17:1753788. doi: 10.3389/fmicb.2026.1753788 (PMC13121134; doi:10.3389/fmicb.2026.1753788)

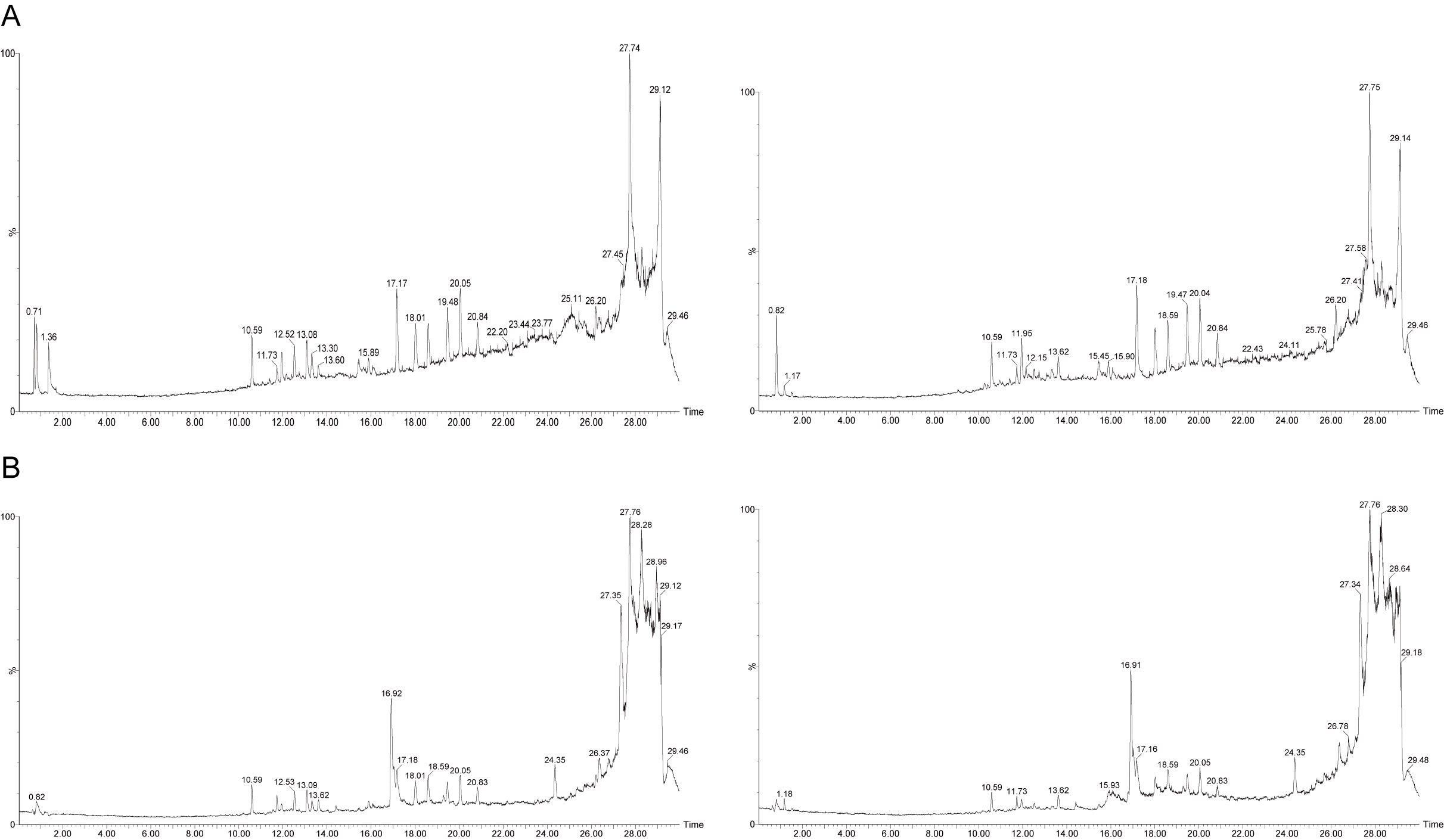

Supplement: SUPPLEMENTARY FIGURE 1 — Profiling of FS and W16 extract via UPLC-Q-TOF/MS. The spectra of FS and W16 in (A) negative ion mode and (B) positive ion mode. [file Image_1.tif]
